# Supplementary material for: How youth cognitive and sociodemographic factors relate to the development of overweight and obesity in the UK and the USA: a prospective cross-cohort study of the National Child Development Study and National Longitudinal Study of Youth 1979
Source: BMJ Open. 2019 Dec 17;9(12):e033011. doi: 10.1136/bmjopen-2019-033011 (PMC6937025; doi:10.1136/bmjopen-2019-033011)
Supplement: Supplementary data [file bmjopen-2019-033011supp001.pdf]

Supplementary Information for

How early-life cognitive and sociodemographic factors relate to the development of overweight and obesity in the UK and the US: a prospective cross-cohort study of the National Child Development Study and National Longitudinal Study of Youth 1979

This file includes:

Appendix 1 – Methods – Income and measurement time
Appendix 2 – Methods – Missing Data
Supplementary Tables S1 to S4

Altschul DM, *et al. BMJ Open* 2019; 9:e033011. doi: 10.1136/bmjopen-2019-033011

Appendix 1

Methods – Income and measurement time

Income variables were taken from the same wave as BMI variables with one exception. In the NCDS, income data were collected using a different instrument at age 55, the most recent available time point for BMI data. The age 55 measurement was missing more data than the age 50 measurement (652 more cases), and in addition to there being data collection inconsistencies at age 55, the variable had some problematic properties, e.g. smaller mean and median income statistics compared to those at age 50. Considering that model fit was sub-par using the age 55 measure, but much better using the age 50 measure, we used the age 50 income measure in all of the analyses we present.

Altschul DM, *et al. BMJ Open* 2019; 9:e033011. doi: 10.1136/bmjopen-2019-033011

Appendix 2

Methods - Missing Data

Study attrition and occasional non-response were present in both samples (see Table 1), though one of the benefits of our approach was that we could grapple with missing data with Full Information Maximum Likelihood estimation using observed information [11]. Standard errors were calculated with first order derivatives and *P*-values were calculated with a χ^2 test, both of which are procedures that can handle missing data.

Prior studies have used multiple imputation to test the robustness of analyses using some of these data [12 13]. They found the same results when comparing imputed and non-imputed model results.

Altschul DM, *et al. BMJ Open* 2019; 9:e033011. doi: 10.1136/bmjopen-2019-033011

Table S1. Full table of parameter estimates from each latent growth curve model of BMI growth in the US and UK.

United States				United Kingdom			
LHS	RHS	Estimate	SE	LHS	RHS	Estimate	SE
Latent Variables							
BMI Level	BMI ('85)	1		BMI Level	BMI (age 23)	1	
BMI Level	BMI ('94)	1		BMI Level	BMI (age 33)	1	
BMI Level	BMI ('06)	1		BMI Level	BMI (age 42)	1	
BMI Level	BMI ('14)	1		BMI Level	BMI (age 55)	1	
BMI Slope	BMI ('85)	0		BMI Slope	BMI (age 23)	0	
BMI Slope	BMI ('94)	0.9		BMI Slope	BMI (age 33)	1	
BMI Slope	BMI ('06)	2.1		BMI Slope	BMI (age 42)	1.9	
BMI Slope	BMI ('14)	2.9		BMI Slope	BMI (age 55)	3.2	
BMI Quadratic	BMI ('85)	0		BMI Quadratic	BMI (age 23)	0	
BMI Quadratic	BMI ('94)	0.81		BMI Quadratic	BMI (age 33)	1	
BMI Quadratic	BMI ('06)	4.41		BMI Quadratic	BMI (age 42)	3.61	
BMI Quadratic	BMI ('14)	8.41		BMI Quadratic	BMI (age 55)	10.24	
Regressions							
BMI Level	Sex (women)	-1.444	0.192	BMI Level	Sex (women)	-0.849	0.085
BMI Level	Cognitive function	0.105	0.166	BMI Level	Cognitive function	-0.196	0.068
BMI Level	Sex x Cognitive function	-0.525	0.192	BMI Level	Sex x Cognitive function	-0.053	0.086
BMI Level	Age ('79)	-0.320	1.399				
BMI Level	Youth SED	0.178	0.115	BMI Level	Youth SED	0.332	0.052
BMI Level	Education	-0.248	0.127	BMI Level	Education	-0.153	0.056
BMI Slope	Sex (women)	0.157	0.198	BMI Slope	Sex (women)	-0.327	0.090
BMI Slope	Cognitive function	0.092	0.177	BMI Slope	Cognitive function	0.036	0.075
BMI Slope	Sex x Cognitive function	0.295	0.195	BMI Slope	Sex x Cognitive function	-0.140	0.093
BMI Slope	Age ('79)	1.754	1.725				

Altschul DM, *et al. BMJ Open* 2019; 9:e033011. doi: 10.1136/bmjopen-2019-033011

BMI Slope	Youth SED	0.281	0.121	BMI Slope	Youth SED	0.028	0.057
BMI Slope	Education	-0.082	0.128	BMI Slope	Education	0.030	0.057
BMI Quadratic	Sex (women)	0.043	0.066	BMI Quadratic	Sex (women)	0.135	0.028
BMI Quadratic	Cognitive function	-0.003	0.059	BMI Quadratic	Cognitive function	-0.009	0.023
BMI Quadratic	Sex x Cognitive function	-0.095	0.065	BMI Quadratic	Sex x Cognitive function	0.026	0.029
BMI Quadratic	Age ('79)	-0.610	0.570				
BMI Quadratic	Youth SED	-0.038	0.041	BMI Quadratic	Youth SED	0.027	0.018
BMI Quadratic	Education	0.020	0.043	BMI Quadratic	Education	-0.015	0.018
BMI ('85)	Age ('85)	1.717	1.358				
BMI ('85)	Income ('85)	-0.098	0.110	BMI (age 23)	Income (age 23)	0.574	0.094
BMI ('94)	Age ('94)	-0.146	1.107				
BMI ('94)	Income ('94)	0.004	0.101	BMI (age 33)	Income (age 33)	-0.185	0.088
BMI ('06)	Age ('06)	-0.664	1.100				
BMI ('06)	Income ('06)	-0.076	0.076	BMI (age 42)	Income (age 42)	0.015	0.047
BMI ('14)	Age ('14)	-0.324	1.584				
BMI ('14)	Income ('14)	-0.142	0.080	BMI (age 55)	Income (age 50)	0.071	0.058
Variances and covariances							
BMI ('85)	BMI ('85)	0.010	0.000	BMI (age 23)	BMI (age 23)	0.010	#N/A
BMI ('94)	BMI ('94)	4.759	0.156	BMI (age 33)	BMI (age 33)	7.578	0.063
BMI ('06)	BMI ('06)	3.603	0.183	BMI (age 42)	BMI (age 42)	2.364	0.087
BMI ('14)	BMI ('14)	5.745	0.514	BMI (age 55)	BMI (age 55)	5.762	0.677
BMI Level	BMI Level	15.309	0.371	BMI Level	BMI Level	9.064	0.124
BMI Slope	BMI Slope	7.299	0.359	BMI Slope	BMI Slope	5.183	0.186
BMI Quadratic	BMI Quadratic	0.436	0.057	BMI Quadratic	BMI Quadratic	0.255	0.034
BMI Level	BMI Slope	-0.319	0.250	BMI Level	BMI Slope	0.384	0.102
BMI Level	BMI Quadratic	-0.002	0.083	BMI Level	BMI Quadratic	-0.071	0.035
BMI Slope	BMI Quadratic	-1.554	0.131	BMI Slope	BMI Quadratic	-1.006	0.071
Sex (women)	Sex (women)	0.250	0.000	Sex (women)	Sex (women)	0.249	0.000

Sex (women)	Cognitive function	-0.041	0.000	Sex (women)	Cognitive function	0.031	0.000
Sex (women)	Sex x Cognitive function	0.052	0.000	Sex (women)	Sex x Cognitive function	0.064	0.000
Sex (women)	Age ('79)	0.003	0.000				
Sex (women)	Youth SED	0.020	0.000	Sex (women)	Youth SED	0.011	0.000
Sex (women)	Education	0.002	0.000	Sex (women)	Education	-0.011	0.000
Sex (women)	Age ('85)	0.004	0.000				
Sex (women)	Income ('85)	-0.017	0.000	Sex (women)	Income (age 23)	0.013	0.000
Sex (women)	Age ('94)	0.003	0.000				
Sex (women)	Income ('94)	-0.015	0.000	Sex (women)	Income (age 33)	0.004	0.000
Sex (women)	Age ('06)	0.004	0.000				
Sex (women)	Income ('06)	-0.045	0.000	Sex (women)	Income (age 42)	-0.025	0.000
Sex (women)	Age ('14)	0.003	0.000				
Sex (women)	Income ('14)	-0.094	0.000	Sex (women)	Income (age 50)	-0.053	0.000
Cognitive function	Cognitive function	0.800	0.000	Cognitive function	Cognitive function	0.862	0.000
Cognitive function	Sex x Cognitive function	0.344	0.000	Cognitive function	Sex x Cognitive function	0.442	0.000
Cognitive function	Age ('79)	0.031	0.000				
Cognitive function	Youth SED	-0.375	0.000	Cognitive function	Youth SED	-0.338	0.000
Cognitive function	Education	0.536	0.000	Cognitive function	Education	0.369	0.000
Cognitive function	Age ('85)	0.031	0.000				
Cognitive function	Income ('85)	0.073	0.000	Cognitive function	Income (age 23)	0.013	0.000
Cognitive function	Age ('94)	0.030	0.000				
Cognitive function	Income ('94)	0.228	0.000	Cognitive function	Income (age 33)	0.103	0.000
Cognitive function	Age ('06)	0.028	0.000				
Cognitive function	Income ('06)	0.353	0.000	Cognitive function	Income (age 42)	0.147	0.000
Cognitive function	Age ('14)	0.030	0.000				
Cognitive function	Income ('14)	0.483	0.000	Cognitive function	Income (age 50)	0.206	0.000
Sex x Cognitive function	Sex x Cognitive function	0.363	0.000	Sex x Cognitive function	Sex x Cognitive function	0.451	0.000
Sex x Cognitive function	Age ('79)	0.014	0.000				

Altschul DM, *et al. BMJ Open* 2019; 9:e033011. doi: 10.1136/bmjopen-2019-033011

Sex x Cognitive function	Youth SED	-0.163	0.000	Sex x Cognitive function	Youth SED	-0.185	0.000
Sex x Cognitive function	Education	0.236	0.000	Sex x Cognitive function	Education	0.176	0.000
Sex x Cognitive function	Age ('85)	0.013	0.000				
Sex x Cognitive function	Income ('85)	0.034	0.000	Sex x Cognitive function	Income (age 23)	0.017	0.000
Sex x Cognitive function	Age ('94)	0.014	0.000				
Sex x Cognitive function	Income ('94)	0.100	0.000	Sex x Cognitive function	Income (age 33)	0.050	0.000
Sex x Cognitive function	Age ('06)	0.012	0.000				
Sex x Cognitive function	Income ('06)	0.159	0.000	Sex x Cognitive function	Income (age 42)	0.070	0.000
Sex x Cognitive function	Age ('14)	0.013	0.000				
Sex x Cognitive function	Income ('14)	0.185	0.000	Sex x Cognitive function	Income (age 50)	0.089	0.000
Age ('79)	Age ('79)	0.049	0.000				
Age ('79)	Youth SED	-0.005	0.000				
Age ('79)	Education	-0.001	0.000				
Age ('79)	Age ('85)	0.049	0.000				
Age ('79)	Income ('85)	0.011	0.000				
Age ('79)	Age ('94)	0.049	0.000				
Age ('79)	Income ('94)	0.012	0.000				
Age ('79)	Age ('06)	0.049	0.000				
Age ('79)	Income ('06)	0.001	0.000				
Age ('79)	Age ('14)	0.048	0.000				
Age ('79)	Income ('14)	-0.004	0.000				
Youth SED	Youth SED	0.760	0.000	Youth SED	Youth SED	0.909	0.000
Youth SED	Education	-0.418	0.000	Youth SED	Education	-0.397	0.000
Youth SED	Age ('85)	-0.006	0.000				
Youth SED	Income ('85)	-0.082	0.000	Youth SED	Income (age 23)	0.012	0.000
Youth SED	Age ('94)	-0.006	0.000				
Youth SED	Income ('94)	-0.176	0.000	Youth SED	Income (age 33)	-0.080	0.000
Youth SED	Age ('06)	-0.004	0.000				

Altschul DM, *et al. BMJ Open* 2019; 9:e033011. doi: 10.1136/bmjopen-2019-033011

Youth SED	Income ('06)	-0.278	0.000	Youth SED	Income (age 42)	-0.151	0.000
Youth SED	Age ('14)	-0.006	0.000				
Youth SED	Income ('14)	-0.378	0.000	Youth SED	Income (age 50)	-0.194	0.000
Education	Education	0.937	0.000	Education	Education	0.982	0.000
Education	Age ('85)	0.000	0.000				
Education	Income ('85)	0.066	0.000	Education	Income (age 23)	-0.033	0.000
Education	Age ('94)	-0.001	0.000				
Education	Income ('94)	0.242	0.000	Education	Income (age 33)	0.100	0.000
Education	Age ('06)	-0.001	0.000				
Education	Income ('06)	0.400	0.000	Education	Income (age 42)	0.180	0.000
Education	Age ('14)	0.000	0.000				
Education	Income ('14)	0.552	0.000	Education	Income (age 50)	0.249	0.000
Age ('85)	Age ('85)	0.050	0.000				
Age ('85)	Income ('85)	0.011	0.000				
Age ('85)	Age ('94)	0.048	0.000				
Age ('85)	Income ('94)	0.013	0.000				
Age ('85)	Age ('06)	0.049	0.000				
Age ('85)	Income ('06)	0.002	0.000				
Age ('85)	Age ('14)	0.048	0.000				
Age ('85)	Income ('14)	-0.003	0.000				
Income ('85)	Income ('85)	0.269	0.000	Income (age 23)	Income (age 23)	0.082	0.000
Income ('85)	Age ('94)	0.011	0.000				
Income ('85)	Income ('94)	0.101	0.000	Income (age 23)	Income (age 33)	0.024	0.000
Income ('85)	Age ('06)	0.011	0.000				
Income ('85)	Income ('06)	0.111	0.000	Income (age 23)	Income (age 42)	0.038	0.000
Income ('85)	Age ('14)	0.011	0.000				
Income ('85)	Income ('14)	0.140	0.000	Income (age 23)	Income (age 50)	0.034	0.000
Age ('94)	Age ('94)	0.050	0.000				

Altschul DM, *et al. BMJ Open* 2019; 9:e033011. doi: 10.1136/bmjopen-2019-033011

Age ('94)	Income ('94)	0.012	0.000				
Age ('94)	Age ('06)	0.048	0.000				
Age ('94)	Income ('06)	0.002	0.000				
Age ('94)	Age ('14)	0.048	0.000				
Age ('94)	Income ('14)	-0.004	0.000				
Income ('94)	Income ('94)	0.450	0.000	Income (age 33)	Income (age 33)	0.287	0.000
Income ('94)	Age ('06)	0.012	0.000				
Income ('94)	Income ('06)	0.388	0.000	Income (age 33)	Income (age 42)	0.155	0.000
Income ('94)	Age ('14)	0.012	0.000				
Income ('94)	Income ('14)	0.446	0.000	Income (age 33)	Income (age 50)	0.170	0.000
Age ('06)	Age ('06)	0.049	0.000				
Age ('06)	Income ('06)	0.001	0.000				
Age ('06)	Age ('14)	0.048	0.000				
Age ('06)	Income ('14)	-0.005	0.000				
Income ('06)	Income ('06)	1.045	0.000	Income (age 42)	Income (age 42)	0.628	0.000
Income ('06)	Age ('14)	0.001	0.000				
Income ('06)	Income ('14)	0.930	0.000	Income (age 42)	Income (age 50)	0.343	0.000
Age ('14)	Age ('14)	0.049	0.000				
Age ('14)	Income ('14)	-0.002	0.000				
Income ('14)	Income ('14)	1.643	0.000	Income (age 50)	Income (age 50)	0.937	0.000
Intercepts							
BMI ('85)		0.000	0.000	BMI (age 23)		0.000	0.000
BMI ('94)		0.000	0.000	BMI (age 33)		0.000	0.000
BMI ('06)		0.000	0.000	BMI (age 42)		0.000	0.000
BMI ('14)		0.000	0.000	BMI (age 55)		0.000	0.000
Sex (women)		0.511	0.000	Sex (women)		0.530	0.000
Cognitive function		0.287	0.000	Cognitive function		0.199	0.000
Sex x Cognitive function		0.106	0.000	Sex x Cognitive function		0.136	0.000

Altschul DM, *et al. BMJ Open* 2019; 9:e033011. doi: 10.1136/bmjopen-2019-033011

Age ('79)	1.807	0.000			
Youth SED	-0.206	0.000	Youth SED	-0.087	0.000
Education	0.303	0.000	Education	0.041	0.000
Age ('85)	2.353	0.000			
Income ('85)	-0.552	0.000	Income (age 23)	-0.961	0.000
Age ('94)	3.297	0.000			
Income ('94)	-0.039	0.000	Income (age 33)	-0.199	0.000
Age ('06)	4.467	0.000			
Income ('06)	0.541	0.000	Income (age 42)	0.349	0.000
Age ('14)	5.347	0.000			
Income ('14)	0.688	0.000	Income (age 50)	0.717	0.000
BMI Level	20.979	1.095	BMI Level	23.645	0.116
BMI Slope	6.103	2.833	BMI Slope	1.929	0.119
BMI Quadratic	-0.822	1.320	BMI Quadratic	-0.186	0.032

LHS = left-hand side; RHS = right-hand side; SED = socioeconomic disadvantage.

Table S2. Coefficient estimates from models of BMI growth, cognitive function, and sociodemographic variables, income excluded.

		United States				United Kingdom			
Outcome	Predictor	Estimate	95%	C.I.	*P*	Estimate	95%	C.I.	*P*
BMI Level	Sex (women)	**-1.572**	**[-1.868,**	**-1.276]**	**< 0.001**	**-0.840**	**[-0.979,**	**-0.701]**	**< 0.001**
	Cognitive function	0.123	[-0.141,	0.387]	0.543	**-0.195**	**[-0.301,**	**-0.089]**	**0.001**
	Sex x Cognitive function	**-0.394**	**[-0.691,**	**-0.097]**	**0.033**	-0.014	[-0.152,	0.123]	0.840
	Youth SED	0.182	[-0.003,	0.367]	0.122	**0.277**	**[0.192,**	**0.363]**	**< 0.001**
	Education	**-0.339**	**[-0.534,**	**-0.143]**	**0.004**	**-0.206**	**[-0.303,**	**-0.110]**	**< 0.001**
BMI Slope	Sex (women)	0.148	[-0.163,	0.459]	0.543	**-0.391**	**[-0.542,**	**-0.239]**	**< 0.001**
	Cognitive function	0.019	[-0.258,	0.296]	0.893	-0.023	[-0.142,	0.096]	0.743
	Sex x Cognitive function	0.257	[-0.049,	0.563]	0.180	-0.118	[-0.270,	0.034]	0.231
	Youth SED	0.209	[0.018,	0.401]	0.096	0.025	[-0.071,	0.122]	0.683
	Education	-0.069	[-0.265,	0.127]	0.627	0.065	[-0.027,	0.157]	0.272
	BMI Level	0.416	[0.012,	0.819]	0.112	0.399	[-0.206,	1.003]	0.294
BMI Quadratic	Sex (women)	0.043	[-0.059,	0.144]	0.567	**0.146**	**[0.098,**	**0.194]**	**< 0.001**
	Cognitive function	0.009	[-0.081,	0.099]	0.893	0.012	[-0.026,	0.050]	0.629
	Sex x Cognitive function	-0.085	[-0.185,	0.016]	0.180	0.020	[-0.030,	0.069]	0.558
	Youth SED	-0.013	[-0.078,	0.052]	0.789	0.027	[-0.004,	0.058]	0.183
	Education	0.014	[-0.059,	0.078]	0.789	-0.025	[-0.053,	0.004]	0.183
	BMI Level	**-0.203**	**[-0.336,**	**-0.071]**	**0.012**	-0.072	[-0.211,	0.067]	0.430
	BMI Slope	**-1.501**	**[-1.724,**	**-1.279]**	**< 0.001**	**-1.041**	**[-1.213,**	**-0.870]**	**< 0.001**
χ2		59.740				254.238			
df		19				6			
CFI		0.996				0.985			
SRMR		0.006				0.015			

Models are latent growth curve models, with latent variables for BMI level, slope, and quadratic slope. The same path diagram was used for both samples, except the US sample was modelled with adjustments for individual ages. All coefficient values are multiple regression coefficients from

the predictor onto the outcome, except the associations between the three BMI latent variables, which are covariances. P-values are corrected for multiple comparisons using the false discovery rate.

Altschul DM, *et al. BMJ Open* 2019; 9:e033011. doi: 10.1136/bmjopen-2019-033011

Table S3. Coefficient estimates from models of BMI growth, cognitive function, and sociodemographic variables, income and education excluded.

		United States				United Kingdom			
Outcome	Predictor	Estimate	95%	C.I.	*P*	Estimate	95%	C.I.	*P*
BMI Level	Sex (women)	**-1.628**	**[-1.922,**	**-1.333]**	**< 0.001**	**-0.926**	**[-1.049,**	**-0.803]**	**< 0.001**
	Cognitive function	-0.060	[-0.306,	0.187]	0.732	**-0.231**	**[-0.321,**	**-0.141]**	**< 0.001**
	Sex x Cognitive function	**-0.400**	**[-0.697,**	**-0.103]**	**0.025**	-0.019	[-0.139,	0.101]	0.810
	Youth SED	**0.274**	**[0.101,**	**0.448]**	**0.010**	**0.343**	**[0.270,**	**0.415]**	**< 0.001**
BMI Slope	Sex (women)	0.146	[-0.163,	0.455]	0.534	**-0.401**	**[-0.546,**	**-0.255]**	**< 0.001**
	Cognitive function	-0.022	[-0.282,	0.238]	0.870	0.026	[-0.085,	0.138]	0.793
	Sex x Cognitive function	0.265	[-0.040,	0.570]	0.151	-0.148	[-0.293,	-0.004]	0.067
	Youth SED	**0.231**	**[0.051,**	**0.412]**	**0.030**	0.018	[-0.068,	0.103]	0.793
	BMI Level	0.386	[-0.017,	0.789]	0.129	**0.330**	**[0.170,**	**0.490]**	**< 0.001**
BMI Quadratic	Sex (women)	0.042	[-0.059,	0.143]	0.565	**0.149**	**[0.102,**	**0.196]**	**< 0.001**
	Cognitive function	0.017	[-0.068,	0.102]	0.749	-0.004	[-0.040,	0.033]	0.849
	Sex x Cognitive function	-0.086	[-0.186,	0.014]	0.151	0.029	[-0.019,	0.077]	0.324
	Youth SED	-0.017	[-0.078,	0.044]	0.732	**0.032**	**[0.004,**	**0.060]**	**0.045**
	BMI Level	**-0.193**	**[-0.326,**	**-0.061]**	**0.016**	-0.058	[-0.114,	-0.001]	0.067
	BMI Slope	**-1.497**	**[-1.718,**	**-1.275]**	**< 0.001**	**-1.076**	**[-1.188,**	**-0.964]**	**< 0.001**
$\chi 2$		58.438				269.375			
df		18				5			
CFI		0.996				0.985			
SRMR		0.006				0.016			

Models are latent growth curve models, with latent variables for BMI level, slope, and quadratic slope. The same path diagram was used for both samples, except the US sample was modeled with adjustments for individual ages. All coefficient values are multiple regression coefficients from the predictor onto the outcome, except the associations between the three BMI latent variables, which are covariances. P-values are corrected for multiple comparisons using the false discovery rate.

Altschul DM, *et al. BMJ Open* 2019; 9:e033011. doi: 10.1136/bmjopen-2019-033011

Table S4. Coefficient estimates from models of BMI growth, intelligence, and sociodemographic variables, using participants from all ethnic groups.

		United States				United Kingdom			
Outcome	Predictor	Estimate	95%	C.I.	*P*	Estimate	95%	C.I.	*P*
BMI Level	Sex (women)	**-1.252**	**[-1.554,**	**-0.950]**	**< 0.001**	**-0.823**	**[-0.983,**	**-0.664]**	**< 0.001**
	Cognitive function	0.133	[-0.109,	0.375]	0.387	**-0.177**	**[-0.304,**	**-0.050]**	**0.015**
	Sex x Cognitive function	**-0.719**	**[-0.994,**	**-0.444]**	**< 0.001**	-0.076	[-0.237,	0.084]	0.503
	Youth SED	**0.268**	**[0.111,**	**0.426]**	**0.003**	**0.322**	**[0.226,**	**0.418]**	**< 0.001**
	Education	-0.169	[-0.349,	0.010]	0.129	**-0.169**	**[-0.273,**	**-0.064]**	**0.004**
BMI Slope	Sex (women)	**0.430**	**[0.112,**	**0.748]**	**0.020**	**-0.320**	**[-0.486,**	**-0.154]**	**< 0.001**
	Cognitive function	-0.216	[-0.471,	0.039]	0.179	0.047	[-0.090,	0.184]	0.606
	Sex x Cognitive function	-0.073	[-0.357,	0.210]	0.792	-0.143	[-0.313,	0.026]	0.180
	Youth SED	**0.437**	**[0.276,**	**0.598]**	**0.000**	0.034	[-0.070,	0.138]	0.606
	Education	0.176	[-0.005,	0.358]	0.124	0.016	[-0.090,	0.122]	0.777
	BMI Level	**0.735**	**[0.290,**	**1.180]**	**0.003**	**0.428**	**[0.238,**	**0.618]**	**< 0.001**
BMI Quadratic	Sex (women)	-0.010	[-0.114,	0.095]	0.913	**0.134**	**[0.083,**	**0.185]**	**< 0.001**
	Cognitive function	0.067	[-0.019,	0.154]	0.215	-0.009	[-0.051,	0.033]	0.752
	Sex x Cognitive function	0.014	[-0.080,	0.108]	0.893	0.025	[-0.029,	0.078]	0.503
	Youth SED	**-0.121**	**[-0.175,**	**-0.067]**	**< 0.001**	0.026	[-0.007,	0.058]	0.216
	Education	-0.037	[-0.097,	0.023]	0.333	-0.012	[-0.046,	0.021]	0.598
	BMI Level	**-0.407**	**[-0.555,**	**-0.259]**	**< 0.001**	**-0.084**	**[-0.147,**	**-0.020]**	**0.021**
	BMI Slope	**-1.938**	**[-2.187,**	**-1.689]**	**< 0.001**	**-0.971**	**[-1.100,**	**-0.840]**	**< 0.001**
χ2		111.284				161.44			
df		31				18			
CFI		0.993				0.990			
SRMR		0.015				0.012			

Models are latent growth curve models, with latent variables for BMI level, slope, and quadratic slope - see the path diagram in Figure 1. The same path diagram was used for both samples, except the US sample was modeled with adjustments for individual ages. All coefficient values are

Altschul DM, *et al. BMJ Open* 2019; 9:e033011. doi: 10.1136/bmjopen-2019-033011

multiple regression coefficients from the predictor onto the outcome, except the associations between the three BMI latent variables, which are covariances. P-values are corrected for multiple comparisons using the false discovery rate. SED = socioeconomic disadvantage.

1. Li W, Kelsey JL, Zhang Z, et al. Small-area estimation and prioritizing communities for obesity control in Massachusetts. *Am J Public Health* 2009;99(3):511-19.
2. Palmer P, Hartke DD, Ree MJ, et al. Armed Services Vocational Aptitude Battery (ASVAB): Alternate Forms Reliability (Forms 8, 9, 10, and 11): AIR FORCE HUMAN RESOURCES LAB BROOKS AFB TX, 1988.
3. Welsh Jr JR, Kucinkas SK, Curran LT. Armed services vocational battery (ASVAB): Integrative review of validity studies: OPERATIONAL TECHNOLOGIES CORP SAN ANTONIO TX, 1990.
4. Der G, Batty GD, Deary IJ. The association between IQ in adolescence and a range of health outcomes at 40 in the 1979 US National Longitudinal Study of Youth. *Intelligence* 2009;37(6):573-80.
5. Wraw C, Deary IJ, Der G, et al. Maternal and offspring intelligence in relation to BMI across childhood and adolescence. *Int J Obesity* 2018 doi: 10.1038/s41366-018-0009-1
6. Herrnstein RJ, Murray C. Bell curve: Intelligence and class structure in American life: Simon and Schuster 2010.
7. Pigeon D. Tests used in the 1954 and 1957 surveys. *The home and the school* 1964:129-32.
8. Douglas JWB. The home and the school: MacGibbon & Kee London 1964.
9. Bridger E, Daly M. Does cognitive ability buffer the link between childhood disadvantage and adult health? *Health Psychol* 2017;36(10):966.
10. Goodman A, Joyce R, Smith JP. The long shadow cast by childhood physical and mental problems on adult life. *Proceedings of the National Academy of Sciences* 2011:201016970.
11. Savalei V. Expected versus observed information in SEM with incomplete normal and nonnormal data. *Psychological methods* 2010;15(4):352.
12. Wraw C, Deary IJ, Gale CR, et al. Intelligence in youth and health at age 50. *Intelligence* 2015;53:23-32.
13. Wraw C, Deary IJ, Der G, et al. Intelligence in youth and mental health at age 50. *Intelligence* 2016;58:69-79.
