# Supplementary material for: How youth cognitive and sociodemographic factors relate to the development of overweight and obesity in the UK and the USA: a prospective cross-cohort study of the National Child Development Study and National Longitudinal Study of Youth 1979
Source: BMJ Open. 2019 Dec 17;9(12):e033011. doi: 10.1136/bmjopen-2019-033011 (PMC6937025; doi:10.1136/bmjopen-2019-033011)
Supplement: Author's manuscript [file bmjopen-2019-033011.draft_revisions.pdf]

BMJ Open is committed to open peer review. As part of this commitment we make the peer review history of every article we publish publicly available.

When an article is published we post the peer reviewers' comments and the authors' responses online. We also post the versions of the paper that were used during peer review. These are the versions that the peer review comments apply to.

The versions of the paper that follow are the versions that were submitted during the peer review process. They are not the versions of record or the final published versions. They should not be cited or distributed as the published version of this manuscript.

BMJ Open is an open access journal and the full, final, typeset and author-corrected version of record of the manuscript is available on our site with no access controls, subscription charges or pay-per-view fees (http://bmjopen.bmj.com).

If you have any questions on BMJ Open's open peer review process please email editorial.bmjopen@bmj.com

BMJ Open

How early-life cognitive and sociodemographic factors relate to the development of overweight and obesity in the UK and the US: a prospective cross-cohort study of the National Child Development Study and National Longitudinal Study of Youth 1979

Journal:	*BMJ Open*
Manuscript ID	bmjopen-2019-033011
Article Type:	Research
Date Submitted by the Author:	16-Jul-2019

Complete List of Authors:	Altschul, Drew; The University of Edinburgh, Psychology<br>Wraw, Christina; National Health Service Scotland<br>Gale, Catharine; MRC Epidemiology Resource Centre<br>Deary, Ian; University of Edinburgh, Department of Psychology

Keywords:	obesity, overweight, longitudinal studies, cognition, socioeconomic factors

SCHOLARONE™
Manuscripts

How early-life cognitive and sociodemographic factors relate to the development of overweight and obesity in the UK and the US: a prospective cross-cohort study of the National Child Development Study and National Longitudinal Study of Youth 1979

Short title: Cognitive and sociodemographic factors and obesity

Drew M Altschul[a,b,c*], Christina Wraw[c,d], Catharine R Gale,[a,c,e] Ian J Deary[a,c]

[a] Department of Psychology, The University of Edinburgh, Edinburgh, EH8 9JZ, UK.

[b] Mental Health Data Science Scotland, Edinburgh, EH8 9JZ, UK.

[c] Centre for Cognitive Ageing and Cognitive Epidemiology, Department of Psychology, University of Edinburgh, Edinburgh, EH8 9JZ, UK.

[d] National Health Service Scotland, Edinburgh, EH12 9EB, UK

[e] MRC Lifecourse Epidemiology Unit, University of Southampton, Southampton, SO16 6YD, UK.

[*]Correspondence to: dmaltschul@gmail.com; 7 George Square, Edinburgh, UK, EH8 9JZ; +44 0131 650 4007

Key words: cognition, socioeconomic factors, obesity, overweight, longitudinal studies

Word count: 3740

For peer review only

ABSTRACT

Objectives

We investigated how early-life cognitive and sociodemographic factors are associated with the etiology of overweight and obesity. We examined both onset (who is at early risk for overweight and obesity), and development (who gains weight and when).

Design

Prospective cohort study.

Setting

We used data from the United States National Longitudinal Study of Youth 1979 (NLSY) and the United Kingdom National Child Development Study (NCDS); most of both studies completed a cognitive function test in youth.

Participants

12,686 and 18,558 members of the NLSY and NCDS, respectively, with data on validated measures of early-life cognitive function, socioeconomic disadvantage, and educational attainment. Height, weight, and income data were available from across adulthood.

Primary and secondary outcome measures

Body Mass Index (BMI) for four time points in individuals' 20s, 30s, 40s, and 50s. We modeled gain in BMI using latent growth curve models to capture linear and quadratic components of change in BMI over time.

Results

For peer review only

Across cultures, higher cognitive function was associated with lower overall BMI. In the UK, 1 standard deviation higher score in cognitive function was associated with a ~0.2 lower BMI (β=-0.20, 95%CI:-0.33 – -0.06, P=0.011). In Americans, this was true only for women (β=-0.53, 95%CI:-0.90 – -0.15, P=0.046), for whom 1 standard deviation higher cognitive function was associated with more than half a point lower BMI. In British participants only, we found limited evidence for negative and positive associations, respectively, between education (β=-0.15, 95%CI:-0.26 – -0.04, P=0.017) and socioeconomic disadvantage (β=0.33, 95%CI:0.23 – 0.43, P<0.001) and higher BMI. Overall, no cognitive or socioeconomic factors were associated with longitudinal changes in BMI.

Conclusions

While sociodemographic and particularly cognitive factors can explain some average weight patterns, differences in who gains weight in adulthood could not be explained by any of these factors.

For peer review only

STRENGTHS AND LIMITATIONS OF THIS STUDY

- This study used two independent cohorts, each with more than 30 years of follow-up, allowing us to model levels and trajectories of BMI cross-culturally.
- The cohorts are representative of the eras and nations they developed in, so our findings are limited to middle-aged Caucasian populations in Western nations.
- Cognitive and sociodemographic variables were available in both samples, and allowed us to compare the associations of these factors with the development and progression of overweight and obesity.
- More work is required to reveal the mechanisms that link cognitive and sociodemographic factors to overweight and obesity.

For peer review only

INTRODUCTION

Obesity is increasingly prevalent worldwide,[1] doubling between 1980 and 2015 in 73 countries, among both children and adults.[2] Obesity and overweight is dangerously prevalent in both the US and UK;[2] in 2015 more than 196,000 deaths in the US and 26,000 deaths in the UK were attributable to overweight and obesity. Furthermore, obesity and overweight are linked to a range of physical ailments, including cardiovascular disease[3], cancer,[4] type II diabetes and osteoarthritis,[5] as well as mental illnesses, such as depression,[6 7] anxiety,[8] and bipolar disorder.[9] It is thus an important issue in public health to identify early life factors that are associated with obesity and overweight in adulthood. To this end, we investigated the relationships of sociodemographics and general cognitive function in youth with the onset and growth of obesity and overweight from the beginning of adulthood into middle age.

From the beginning of life, inherent biological factors such as sex play a role in determining bodily health,[10] quantified through measures like body mass index (BMI), which is used to define who is overweight and obese. BMI is estimated at between 40% and 70% heritable.[11] On the other hand, early life socioeconomic disadvantage (SED) and adverse experiences are associated with higher risk of overweight and obesity,[12 13] though these findings are confounded by ethnic and geographic disparities.[14 15]

Cognitive function is also an important predictor of both physical and mental health outcomes,[16-21] and lower cognitive function appears to precede obesity.[22-24] Early-life, pre-morbid cognitive function in particular is the gold standard for cognitive epidemiological studies that seek to investigate the long-term associations between cognitive function and the development of disease. An early, pre-morbid measure of cognitive function can avoid confounding. Cognitive function declines on average with age[25] and in the presence of disease comorbidity.[26-28] Moreover, obesity itself has been linked to high incidence of

For peer review only - http://bmjopen.bmj.com/site/about/guidelines.xhtml

Alzheimer's,[29] Parkinson's,[30] and other sources of cognitive decline and dementia in older age.[31] Therefore, early-life cognitive function measures are especially important for understanding the relationships between cognitive function and overweight and obesity.

Gradients in SED affect women's physical health differently from men's, such that greater SED in women is more consistently associated with poorer health.[32 33] Therefore, might gradients in cognitive function also affect women's health similarly, i.e. with stronger associations between cognitive function and health than in men? In 1996, obesity was more likely in English women who were more disadvantaged, e.g. had lower occupational status, but no such association was present in English men.[32] In related health conditions such as diabetes,[33] heart disease,[34 35] and hypertension,[36] women coming from greater disadvantage also had poorer disease outcomes. However, associations between early-life SED and health might be at least partially confounded by differential effects of early life cognitive function, which are also associated with later life health outcomes, including overweight and obesity.[37] Cognitive function is an important predictor of later life health in both sexes, but it plays a stronger role in women. Evidence of this has been found in both the US[38] and UK,[35 37] for high blood pressure, stroke, and coronary heart disease.

Our main goal in the present study was to compare, between the US and UK, which major early-life influences are associated with BMI from young adulthood to middle age. In particular, we wished to investigate the role of general cognitive function and its potential interaction with sex, in BMI changes from early adulthood to middle age. To this end, we analyzed two large, comparable samples from the US and UK. They are, respectively, the National Longitudinal Study of Youth 1979 (NLSY) and the National Child Development Study (NCDS). Both studies have followed participants from youth, when a test of general cognitive function was given. Sociodemographic variables from youth were also available, allowing us to consider other known factors that contribute to overweight and obesity. BMI

For peer review only - http://bmjopen.bmj.com/site/about/guidelines.xhtml

was tracked longitudinally from participants' 20s into their 50s, allowing us to model its growth curves and determine which early-life cognitive and sociodemographic factors are associated with higher BMI over the course of adulthood.

METHODS

The National Longitudinal Study of Youth 1979 (NLSY)

The NLSY was initially sampled from non-institutionalized young people aged 14 – 21 years and living in the US at the beginning of 1979. The original sample consisted of 12,686 participants, and was ethnically representative of the US at that time; 16% of participants were "Hispanic", 25% were "black", and 59% were "non-Black non-Hispanic" – 7506 individuals. The initial interview took place in 1979, and respondents were regularly re-interviewed up to 2014, which is when the most recent data were available. 3396 individual participated in giving height and weight measurements in 2014 (Table 1). The ethnic categories were less specific than in the NCDS, so for example, some Asian-Americans were likely included as "non-Black non-Hispanic", though in 1980 only 1.5% of the US population was of Asian or Pacific Islander descent.

The National Child Development Study (NCDS)

Participants of the NCDS were born in the UK during one week in March 1958. As originally intended, the NCDS was designed to study stillbirth and death in infancy. Later developments in the survey have resulted in multiple follow-ups at regular intervals. Eleven waves of data have been collected, the first at birth and the most recent in 2013, when participants were 55 years old. Of the initial 18,558 participants, 12,440 were "Euro-

For peer review only - http://bmjopen.bmj.com/site/about/guidelines.xhtml

Caucasian". By the 2013 wave, 6861 individuals participated in giving height and weight measurements, again see Table 1.

Variables

BMI and income were assessed 4 times, when the average age was 24, 33, 45, and 53 in the NLSY, and 23, 33, 42, and 55 in the NCDS. Income was measured at the same ages, except for the NCDS, where income was measured at age 50 instead of 55. Education was measured as highest grade achieved, in the NLSY, and the age at which in individual left school, in the NCDS. SED and general cognitive function were both measured early in life, between the ages of 15 and 22 in the NLSY[39] and at age 11 in the NCDS.[40] Additional details on these variables is available in Appendix 1 of the Supplementary Information.

Latent Growth Curve Modeling

Latent growth curve models are a form of structural equation model[41] where, in this case, the latent variables are derived from 4 BMI measurements taken at roughly decade-long intervals. The BMI latent variables included variables for overall BMI level, linear slope, and quadratic slope. All analyses were carried out using the R package 'lavaan'[42].

Youth SED, education, sex, cognitive function, and the interaction between sex and cognitive function were regressed on each of these BMI latent variables. Income for each time point was regressed directly onto the BMI measurements. Because there was substantially more age variation among NLSY participants, additional control variables were included in those models. These were age at cognitive testing in 1979, which was regressed upon the BMI latent variables like cognitive function, and age during each follow-up wave,

For peer review only - http://bmjopen.bmj.com/site/about/guidelines.xhtml

which was regressed upon the BMI measurements like income. The variances of the first BMI observations had to be constrained to be greater than 0 in both the NLSY and NCDS models, but this did not substantially impact the fit of either model. Missing data handling procedures are described in Appendix 2 of the Supplementary Information.

Patient and public involvement

Data were deidentified and no patients were involved in this research. The results of this project will be disseminated via the usual academic media and in the press.

RESULTS

Background

American participants from the NLSY were born between 1956 and 1964. British participants from the NCDS were all born in 1958. The majority of individuals with identifiable ethnicity in the NCDS were 10953 “Euro-Caucasian(s)” – only 465 other participants could have their ethnicity identified. The majority of NLSY participants were identified as 7506 “non-Black non-Hispanic(s)”. 5172 NLSY participants were either Black or Hispanic. Analyses were limited to only non-minority ethnic group individuals to allow between-country comparisons.[43 44] There were not enough minority participants in the NCDS to carry out cross-national analyses on ethnic minority participants.

We modeled change in BMI across time using latent growth curve models. This type of growth curve model fits latent variables to observed variables, with the latent variables representing an individual’s overall level (intercept) of a variable across time, as well as

For peer review only - http://bmjopen.bmj.com/site/about/guidelines.xhtml

latent variables for change (slope, or trajectory) over time. These latent variables can be related to other observed variables with conventional regression and covariance methods.

All models were a variation on the one presented in Figure 1. BMI change over time was best modeled with quadratic and linear slope components in both samples (NLSY: χ^2 = 30.743, df = 13, CFI = 0.998, SRMR = 0.009; NCDS: χ^2 = 286.905, df = 1, CFI = 0.985, SRMR = 0.027), suggesting that BMI increases over time, and that the rate of increase generally decreases over time (Table S1). In our main models, we included effects of education, youth SED, cognitive function in youth, sex, and a sex by cognitive function interaction on the latent variables of BMI level, BMI linear slope, and BMI quadratic slope. Income corresponding to each time point was regressed on the BMI measurement at that point, as was age at measurement in the NLSY. The main results of our models are shown in Table 2, and explained below. All *P* values are two-side and have been corrected for multiple comparisons using the False Discovery Rate[45] within models.

Overview of the results

We first describe the general appearance of the results, before reporting the results of the models in each country separately. In both the US and the UK, and in men and women, mean BMI increases from the 20s to the 50s, by about 4 to 5 BMI points (Table 1, Figure 2). Men appear to have higher mean BMI than women (Figure 2, upper panel). Americans have higher BMI than the British from early adulthood, and the differences grow as adulthood advances; Americans were more than half a BMI point higher than the British when participants were in their 20s, and nearly 1.5 BMI points higher when participants were in their 40s. Although IQ was modelled as a continuum, we show it in Figure 2 as tertiles for descriptive purposes only. In both the US and the UK, throughout the period of adulthood

For peer review only - http://bmjopen.bmj.com/site/about/guidelines.xhtml

examined, the highest IQ tertile has the lowest mean BMI and the lowest IQ tertile has the highest BMI (Figure 2, middle panel). This was seen in both countries, and in men and women (Figure 2, lower panel). Next, we report the results of formal modelling of that data.

United States

In the US sample, men had higher BMI level than women, but there were no main effects of cognitive function, early life sociodemographic characteristics, or education (Table 2). There was evidence for an interaction between sex and cognitive function on BMI; the model's results suggest that cognitive function is not related to BMI level in American men, but, in American women, 1 standard deviation higher cognitive function is associated with a more than half point lower BMI level.

We found no associations between early-life cognitive function or sociodemographic variables and BMI growth, although linear BMI change was negatively associated with quadratic BMI change. This suggests more rapid BMI growth earlier in life is accompanied by a flattening-out of growth in middle-age, which is consistent with what we see in our visualizations (Figure 2).

United Kingdom

In the British sample, women had lower BMI levels than men, more than 0.8 points all else being equal (Table 2). Higher cognitive function in youth was associated with lower BMI level; 1 standard deviation higher score in cognitive function was associated with ~0.2 points lower BMI. There was no sex by cognitive function interaction on BMI level. Additionally, and unlike in the American sample, high youth SED and more educational

For peer review only - http://bmjopen.bmj.com/site/about/guidelines.xhtml

attainment were associated with higher and lower BMI levels, respectively. We note that these coefficients in the US samples are not dissimilar to those in the UK sample, and that the UK sample has greater power.

As found in the American sample, there were no relationships between BMI growth and cognitive function, youth SED, or education. However, there was an association between BMI growth and sex, so that women had slower growth in BMI over time. Every decade, men gained about 0.3 BMI points more than women, all else being equal. Sex was also associated with the quadratic component of BMI growth, such that men's BMI growth tended to level off in later ages, whereas women's linear BMI growth continued more linearly in later ages. These British effects are visible in Figure 2.

In the UK sample, BMI level was associated with linear BMI slope, and linear slope was associated with quadratic slope. This means that higher BMI level indicated greater linear growth, and having a higher linear growth component was associated with more flattening out of growth over time. At one point in time, and only in the British sample, income was related to BMI: at age 23, having a higher income was associated with having higher BMI at that age (B = 0.574, 95% C.I.: 0.390, 0.758, P < 0.001).

Sensitivity Analyses

As our primary interest was in early-life factors, we wished to explore how removing other variables, first income, then education, might influence associations with BMI variables. Moreover, some of these income and education measurements are missing for some participants, so fitting models without them would give us additional power to detect effects, by not requiring that our models rely as heavily on maximum likelihood estimation to fill in the missing values.

For peer review only - http://bmjopen.bmj.com/site/about/guidelines.xhtml

Modeling without income (Table S2) allowed us to confirm the association between higher education and lower BMI levels in the US (B = -0.339, 95% C.I.: -0.534, -0.143, P = 0.004). Associations were unchanged in the UK when income was removed. Going further and removing education (Table S3) revealed associations in the US between SED in youth and BMI level (B = 0.274, 95% C.I.: 0.101, 0.448, P = 0.010) and BMI slope (B = 0.231, 95% C.I.: 0.051, 0.412, P = 0.030), such that more disadvantaged Americans have higher overall BMI levels and faster growth. Removing education in the UK model revealed an association between youth SED and quadratic BMI growth (B = 0.032, 95% C.I.: 0.004, 0.060, P = 0.045), which would suggest that all else being equal, more disadvantaged British participants showed less leveling off of BMI growth over time.

DISCUSSION

From their 20s to their 50s, both the US and UK participants had similar starting points and trajectories of growth in BMI. Almost everyone gained weight as they aged. Both populations show steeper BMI growth in their 20s and 30s, and neither sociodemographic nor cognitive factors appeared to prevent this. Nevertheless, some factors did predict whether participants would have lower or higher BMI levels that would be reflected from early adulthood into middle age, though these factors often differed between the American and the British samples.

A straightforward source of difference in BMI levels came from sex. Both American and British women have lower BMIs than men. However, American women are likely to have nearly 1.5 fewer BMI points than American men, whereas British women are likely to have only about 0.85 fewer BMI points than British men. A possible reason is visible in

For peer review only - http://bmjopen.bmj.com/site/about/guidelines.xhtml

Figure 2: because Americans weigh more in general and there is a greater range of BMI, there is more opportunity for American men to have higher BMI.

In their 20s, the lowest BMI American women approximately matched the lowest BMI British women, but the American sample rapidly gained more weight, and by their 40s, the average American is more than a full BMI point higher. Diversity in BMI grows across both samples as well: the SD of BMI among Americans goes from 4 points in the early 20s to more than 6 in the 50s, and among the British the SD rises from ~3 at age 23 to ~5.5 at age 55. BMI growth is faster in British men than British women, but that growth slowed in participants' 40s and 50s, whereas in British women, growth was slower but more consistent. No such effects were apparent in Americans. Both men and women tended to gain weight at the same rate.

Associations between cognitive function and BMI levels were present in both samples. British with higher cognitive function tended to have lower BMI: a British person of average height (~1.7m) with a 15 IQ point (1SD) lower score than average would likely weigh at least half a kilogram (1.1 lbs.) more than average. On the other hand, only higher cognitive function American women were likely to have lower BMI. For an average American woman (~1.6m), a 15 IQ point lower score than average would likely be equated with being 1.3 kilogram heavier (2.9 lbs.). Cognitive function was one of the more noticeable sources of difference in BMI (Figure 2); in the American sample, only cognitive function and sex, not socioeconomic factors, appeared to be associated with BMI.

In addition to cognitive function, education and youth SED were also associated with BMI levels in British people, but not in Americans. Having more education was associated with lower BMI, to nearly the same extent as cognitive function. Coming from a less deprived background was also associated with lower BMI, to a greater degree than either

For peer review only - http://bmjopen.bmj.com/site/about/guidelines.xhtml

cognitive function or education, though, again, only in the British. In the American sample, education showed a similar effect size and a nearly overlapping confidence interval; therefore, with more power, we would have likely found that education was similarly associated in the American sample. Income was not associated with BMI at any point in our analyses, except among the British when they were age 23. At this age, income may be related to BMI in the same way as youth SED, which is composed of parents' sociodemographic variables from a few years earlier.

Particular strengths of this study include that it is longitudinal, with multiple measurements spanning 30 years. Moreover, a valuable and unusual strength is that the variables and their life-course timings in the two countries were remarkably similar. We identified and derived highly analogous variables for every major factor in the study, making the direct comparisons across these two cultures feasible.

There were some limitations to this study as well. For greater comparability between samples, we studied American and British participants of European descent. This gave our analyses greater clarity, but it reduces the generalizability. Particularly in the US, a major portion of the population has some non-European ancestry, and this proportion is growing in both the US and UK. However, previous analyses give us no reason to believe that these findings are not applicable in other ethnic groups.[19 38] The NLSY and NCDS samples are now both middle-aged, so our growth curve models may not fit in precisely the same way in other age groups, particularly younger samples, whose early-life background may have been different from what our participants experienced in the 1950s, 60s, and 70s. Additionally, our American sample was smaller than our British sample, so we did not have as much power to detect relationships in Americans.

For peer review only

For peer review only - http://bmjopen.bmj.com/site/about/guidelines.xhtml

Previous work with the NLSY has demonstrated that trajectories of BMI in adulthood are associated with social factors including sex, SED, and education.[46 47] Cognitive function in youth has also been linked to maternal and offspring BMI in the NLSY,[20] but to the best of our knowledge, cognitive function has not been associated with trajectories of BMI across the life course. Similarly, studies with British cohorts, including the NCDS, have found that socioeconomic disparities are associated with BMI differences,[48 49] but cognitive function and trajectories of BMI is much less studied. A notable exception is Chandola, et al. [37] who also used LGCMs with the NCDS, but analyzed men and women in different models, thus could not detect the interaction effects we found. Our use of LGCMs allowed us to model change in individuals' BMI over the same period of time, incorporating the same variables, but in distinct cultures. Moreover, our analyses being are cross-cultural provides further validation for our results by demonstrating that they are not unique to a particular nation or culture.

What is apparent is from our results is that cognitive function tested in youth has an important association with BMI, from early adulthood into middle-age. BMI and cognitive function are genetically correlated at r_g = -0.13, [50] so some of the association we found might be caused by genetic variation; however, some of the genetic risk of obesity can be reduced through education.[51] It is therefore important to ask: do people learn things over the course of their education that they use to live healthier lives? In much the same way, we might be able to encourage what has been termed "phenocopying"[17] – that is, encouraging and enabling people to follow the same strategies that individuals with higher cognitive function use to look after their health with the hope of achieving the same results. In contrast to education, which we only found to be associated with BMI in the UK, we found associations between cognitive function and BMI in both samples, as did two prior studies.[50 51]

The associations between cognitive function, sociodemographic factors, and BMI were mostly with an individual's overall level of BMI. BMI level is thus of particular

For peer review only - http://bmjopen.bmj.com/site/about/guidelines.xhtml

importance because it indicates whether a person is overweight or obese from the start of these samples, and obesity among younger adults is particularly hazardous to one's later life health.[52] Youth SED might be linked to major early life determinants of overweight and obesity,[53] and so, through phenocopying and related health education, early interventions are probably best positioned to make a difference to individuals' lifelong health.

This study shows that BMI growth is similar across two nationalities, in spite of major differences in BMI baselines between the US and UK. Moreover, BMI growth appears to be largely unaffected by cognitive and sociodemographic factors. Baseline BMI levels (rather than their rate of change) are influenced by external factors - cognitive function being one of the strongest; its effect appeared in both the US and UK samples. Identifying the behaviors and other pathways through which cognitive function might protect individuals from overweight and obesity should be investigated further in the pursuit of real-world solutions that can help us reduce the global burden of the overweight and obesity epidemic.

AUTHORSHIP CONTRIBUTIONS

DMA discussed and planned the study and analyses, analysed the data, interpreted the data, and drafted the initial manuscript. CW discussed and planned the study and analyses, interpreted the data and contributed to the manuscript. CRG discussed and planned the study and analyses, interpreted the data and contributed to the manuscript. IJD discussed and planned the study and analyses, interpreted the data and contributed to the manuscript.

For peer review only - http://bmjopen.bmj.com/site/about/guidelines.xhtml

DATA SHARING STATEMENT

Anonymized NCDS data are available from the UK Data Service at https://www.ukdataservice.ac.uk/. Anonymized NLSY data are available online from the US Bureau of Labor Statistics at https://www.nlsinfo.org/investigator/.

FUNDING STATEMENT

This work was supported by the University of Edinburgh Centre for Cognitive Ageing and Cognitive Epidemiology, part of the cross council Lifelong Health and Wellbeing Initiative (MR/K026992/1). Funding from the Biotechnology and Biological Sciences Research Council (BBSRC), Economic and Social Research Council (ESRC) and Medical Research Council (MRC) is gratefully acknowledged. This work was also supported by an MRC Mental Health Data Pathfinder award (MC_PC_17209).

COMPETING INTERESTS

All authors declare that they have no competing interests.

For peer review only

References

1. Roberto CA, Swinburn B, Hawkes C, et al. Patchy progress on obesity prevention: emerging examples, entrenched barriers, and new thinking. *The Lancet* 2015;385(9985):2400-09.

2. Collaborators GO. Health effects of overweight and obesity in 195 countries over 25 years. *New Engl J Med* 2017;377(1):13-27.

3. Bogers RP, Bemelmans WJ, Hoogenveen RT, et al. Association of overweight with increased risk of coronary heart disease partly independent of blood pressure and cholesterol levels: a meta-analysis of 21 cohort studies including more than 300 000 persons. *Archives of internal medicine* 2007;167(16):1720-28.

4. Renehan AG, Tyson M, Egger M, et al. Body-mass index and incidence of cancer: a systematic review and meta-analysis of prospective observational studies. *The Lancet* 2008;371(9612):569-78.

5. Guh DP, Zhang W, Bansback N, et al. The incidence of co-morbidities related to obesity and overweight: a systematic review and meta-analysis. *BMC Public Health* 2009;9(1):88.

6. Luppino FS, de Wit LM, Bouvy PF, et al. Overweight, obesity, and depression: a systematic review and meta-analysis of longitudinal studies. *Archives of general psychiatry* 2010;67(3):220-29.

7. De Wit L, Luppino F, van Straten A, et al. Depression and obesity: a meta-analysis of community-based studies. *Psychiatry research* 2010;178(2):230-35.

8. Gariepy G, Nitka D, Schmitz N. The association between obesity and anxiety disorders in the population: a systematic review and meta-analysis. *Int J Obesity* 2010;34(3):407.

9. Zhao Z, Okusaga OO, Quevedo J, et al. The potential association between obesity and bipolar disorder: a meta-analysis. *Journal of affective disorders* 2016;202:120-23.

10. Heid IM, Jackson AU, Randall JC, et al. Meta-analysis identifies 13 new loci associated with waist-hip ratio and reveals sexual dimorphism in the genetic basis of fat distribution. *Nat Genet* 2010;42(11):949.

11. Farooqi I, O'Rahilly S. New advances in the genetics of early onset obesity: Nature Publishing Group, 2005.

12. Shrewsbury V, Wardle J. Socioeconomic status and adiposity in childhood: a systematic review of cross-sectional studies 1990–2005. *Obesity* 2008;16(2):275-84.

13. Danese A, Tan M. Childhood maltreatment and obesity: systematic review and meta-analysis. *Mol Psychiatry* 2014;19(5):544.

14. Taveras EM, Gillman MW, Kleinman K, et al. Racial/Ethnic Differences in Early-Life Risk Factors for Childhood Obesity. *Pediatrics* 2010;125(4):686.

15. Wang Y, Beydoun MA. The obesity epidemic in the United States—gender, age, socioeconomic, racial/ethnic, and geographic characteristics: a systematic review and meta-regression analysis. *Epidemiol Rev* 2007;29(1):6-28.

16. Koenen KC, Moffitt TE, Roberts AL, et al. Childhood IQ and adult mental disorders: a test of the cognitive reserve hypothesis. *Am J Psychiatry* 2009;166(1):50-57.

17. Deary IJ, Weiss A, Batty GD. Intelligence and personality as predictors of illness and death: How researchers in differential psychology and chronic disease epidemiology are collaborating to understand and address health inequalities. *Psychol Sci Publ Int* 2010;11:53-79. doi: 10.1177/1529100610387081

18. Calvin CM, Batty GD, Der G, et al. Childhood intelligence in relation to major causes of death in 68 year follow-up: prospective population study. *bmj* 2017;357:j2708.

19. Wraw C, Deary IJ, Gale CR, et al. Intelligence in youth and health at age 50. *Intelligence* 2015;53:23-32.

20. Wraw C, Deary IJ, Der G, et al. Maternal and offspring intelligence in relation to BMI across childhood and adolescence. *Int J Obesity* 2018 doi: 10.1038/s41366-018-0009-1

21. Wraw C, Deary IJ, Der G, et al. Intelligence in youth and mental health at age 50. *Intelligence* 2016;58:69-79.

For peer review only

For peer review only - http://bmjopen.bmj.com/site/about/guidelines.xhtml

22. Kanazawa S. Intelligence and obesity: which way does the causal direction go? *Current Opinion in Endocrinology, Diabetes and Obesity* 2014;21(5):339-44.

23. Batty G, Gale C, Mortensen LH, et al. Pre-morbid intelligence, the metabolic syndrome and mortality: the Vietnam Experience Study. *Diabetologia* 2008;51(3):436-43.

24. Belsky DW, Caspi A, Goldman-Mellor S, et al. Is obesity associated with a decline in intelligence quotient during the first half of the life course? *Am J Epidemiol* 2013;178(9):1461-68.

25. Verhaeghen P, Salthouse TA. Meta-analyses of age–cognition relations in adulthood: Estimates of linear and nonlinear age effects and structural models. *Psychological bulletin* 1997;122(3):231.

26. Cheng G, Huang C, Deng H, et al. Diabetes as a risk factor for dementia and mild cognitive impairment: a meta-analysis of longitudinal studies. *Intern Med J* 2012;42(5):484-91. doi: 10.1111/j.1445-5994.2012.02758.x

27. Lang CC, Mancini DM. Non-cardiac comorbidities in chronic heart failure. *Heart* 2007;93(6):665-71.

28. Snyder HR. Major depressive disorder is associated with broad impairments on neuropsychological measures of executive function: a meta-analysis and review. *Psychological bulletin* 2013;139(1):81.

29. Profenno LA, Porsteinsson AP, Faraone SV. Meta-analysis of Alzheimer's disease risk with obesity, diabetes, and related disorders. *Biological psychiatry* 2010;67(6):505-12.

30. Chen J, Guan Z, Wang L, et al. Meta-analysis: overweight, obesity, and Parkinson's disease. *International journal of endocrinology* 2014;2014

31. Beydoun MA, Beydoun H, Wang Y. Obesity and central obesity as risk factors for incident dementia and its subtypes: a systematic review and meta-analysis. *Obesity reviews* 2008;9(3):204-18.

32. Wardle J, Waller J, Jarvis MJ. Sex differences in the association of socioeconomic status with obesity. *Am J Public Health* 2002;92(8):1299-304.

33. Kavanagh A, Bentley RJ, Turrell G, et al. Socioeconomic position, gender, health behaviours and biomarkers of cardiovascular disease and diabetes. *Social science & medicine* 2010;71(6):1150-60.

34. Thurston RC, Kubzansky LD, Kawachi I, et al. Is the association between socioeconomic position and coronary heart disease stronger in women than in men? *Am J Epidemiol* 2005;162(1):57-65.

35. Lawlor DA, Batty GD, Clark H, et al. Association of childhood intelligence with risk of coronary heart disease and stroke: findings from the Aberdeen Children of the 1950s cohort study. *Eur J Epidemiol* 2008;23(10):695-706.

36. Leng B, Jin Y, Li G, et al. Socioeconomic status and hypertension: a meta-analysis. *Journal of hypertension* 2015;33(2):221-29.

37. Chandola T, Deary IJ, Blane D, et al. Childhood IQ in relation to obesity and weight gain in adult life: the National Child Development (1958) Study. *Int J Obesity* 2006;30(9):1422.

38. Altschul DM, Wraw C, Der G, et al. Hypertension development by midlife and the roles of pre-morbid cognitive function, sex, and their interaction. *Hypertension* 2019

39. Rothstein DS, Carr D, Cooksey E. Cohort Profile: The National Longitudinal Survey of Youth 1979 (NLSY79). *Int J Epidemiol* 2018 doi: https://doi.org/10.1093/ije/dyy133

40. Pigeon D. Tests used in the 1954 and 1957 surveys. *The home and the school* 1964:129-32.

41. Preacher KJ. Latent growth curve modeling: Sage 2008.

42. Rosseel Y. lavaan: an R package for structural equation modeling and more Version 0.4-9 (BETA): Ghent University, 2011.

43. Bell CG, Walley AJ, Froguel P. The genetics of human obesity. *Nature reviews genetics* 2005;6(3):221.

For peer review only - http://bmjopen.bmj.com/site/about/guidelines.xhtml

44. Carmienke S, Freitag M, Pischon T, et al. General and abdominal obesity parameters and their combination in relation to mortality: a systematic review and meta-regression analysis. *European journal of clinical nutrition* 2013;67(6):573.
45. Benjamini Y, Hochberg Y. Controlling the false discovery rate: A practical and powerful approach to multiple testing. *Journal of the Royal Statistical Society: Series B (Methodological)* 1995;57:289-300.
46. Østbye T, Malhotra R, Landerman LR. Body mass trajectories through adulthood: results from the National Longitudinal Survey of Youth 1979 Cohort (1981–2006). *Int J Epidemiol* 2010;40(1):240-50. doi: 10.1093/ije/dyq142
47. Benson R, von Hippel PT, Lynch JL. Does more education cause lower BMI, or do lower-BMI individuals become more educated? Evidence from the National Longitudinal Survey of Youth 1979. *Social Science & Medicine* 2018;211:370-77.
48. Bann D, Johnson W, Li L, et al. Socioeconomic Inequalities in Body Mass Index across Adulthood: Coordinated Analyses of Individual Participant Data from Three British Birth Cohort Studies Initiated in 1946, 1958 and 1970. *PLOS Medicine* 2017;14(1):e1002214. doi: 10.1371/journal.pmed.1002214
49. Bann D, Johnson W, Li L, et al. Socioeconomic inequalities in childhood and adolescent body-mass index, weight, and height from 1953 to 2015: an analysis of four longitudinal, observational, British birth cohort studies. *The Lancet Public Health* 2018;3(4):e194-e203. doi: https://doi.org/10.1016/S2468-2667(18)30045-8
50. Davies G, Lam M, Harris SE, et al. Study of 300,486 individuals identifies 148 independent genetic loci influencing general cognitive function. *Nature communications* 2018;9(1):2098.
51. Barcellos SH, Carvalho LS, Turley P. Education can reduce health differences related to genetic risk of obesity. *Proceedings of the National Academy of Sciences* 2018;115(42):E9765.
52. Fontaine KR, Redden DT, Wang C, et al. Years of life lost due to obesity. *JAMA* 2003;289(2):187-93. doi: 10.1001/jama.289.2.187
53. Monasta L, Batty G, Cattaneo A, et al. Early-life determinants of overweight and obesity: a review of systematic reviews. *Obesity reviews* 2010;11(10):695-708.

For peer review only

For peer review only - http://bmjopen.bmj.com/site/about/guidelines.xhtml

527 **Figure 1. Path diagram of latent growth curve models used in primary analyses.** Square boxes are observed variables, circles are latent variables. Single headed arrows are regressions or latent variable loadings, double headed arrows are covariances.

531 **Figure 2. Quadratic growth curves of BMI fitted to tertiles of cognitive function, and across different sexes.** The top panels illustrates BMI between sexes, the middle panels illustrates BMI in different general cognitive function (g) tertiles, and the bottom panels illustrate the interaction between cognitive function tertiles and sex. Shaded areas represent the 95% error regions.

For peer review only

For peer review only - http://bmjopen.bmj.com/site/about/guidelines.xhtml

Table 1. Descriptive statistics for all tested variables.

	NLSY - United States				NCDS - United Kingdom			
Variable		N	Median	SD		N	Median	SD
BMI	average age				average age			
	24	6198	22.71	4.00	23	9835	22.13	3.09
	33	4254	24.96	4.98	33	8741	24.62	4.57
	45	3651	26.62	5.53	42	8787	25.43	4.66
	53	3396	27.77	6.04	55	6861	26.79	5.49
Income								
	24	5386	$382	$365.17	23	10027	£87	£52.25
	33	3709	$769	$766.83	33	9094	£272	£178.64
	45	3593	$1288	$1624.87	42	9032	£435	£399.02
	53	3092	$1415	$2133.50	50	7846	£600	£554.46
Cognitive function		7023	0.12			11571	0.13	
Youth SED		7023	0.01			12290	0.15	
Education		3788	-0.03			8819	-0.53	
Sex			3787 male	3719 female			5633 male	5320 female

NLSY = National Longitudinal Study of Youth 1979, NCDS = National Child Development Study, SED = Socioeconomic Disadvantage. Cognitive function, youth SED, and education, are unitless variables, thus they are scaled so that their SD is 1. The means are 0, so the medians indicate the amount of skewness in the distributions.

For peer review only - http://bmjopen.bmj.com/site/about/guidelines.xhtml

Table 2. Coefficient estimates from models of BMI growth, cognitive function, and sociodemographic variables.

		United States				United Kingdom			
Outcome	Predictor	Estimate	95%	C.I.	*P*	Estimate	95%	C.I.	*P*
BMI Level	Sex (women)	**-1.444**	**[-1.820,**	**-1.068]**	**< 0.001**	**-0.849**	**[-1.016,**	**-0.681]**	**< 0.001**
	Cognitive function	0.105	[-0.221,	0.431]	0.683	**-0.196**	**[-0.330,**	**-0.063]**	**0.011**
	Sex x Cognitive function	**-0.525**	**[-0.901,**	**-0.149]**	**0.046**	-0.053	[-0.222,	0.116]	0.694
	Youth SED	0.178	[-0.047,	0.403]	0.348	**0.332**	**[0.230,**	**0.434]**	**< 0.001**
	Education	-0.248	[-0.497,	0.001]	0.225	**-0.153**	**[-0.263,**	**-0.042]**	**0.017**
BMI Slope	Sex (women)	0.157	[-0.230,	0.544]	0.670	**-0.327**	**[-0.504,**	**-0.150]**	**0.001**
	Cognitive function	0.092	[-0.254,	0.438]	0.733	0.036	[-0.110,	0.182]	0.694
	Sex x Cognitive function	0.295	[-0.087,	0.677]	0.348	-0.140	[-0.322,	0.041]	0.239
	Youth SED	0.281	[0.045,	0.518]	0.108	0.028	[-0.084,	0.140]	0.694
	Education	-0.082	[-0.332,	0.168]	0.683	0.030	[-0.082,	0.143]	0.694
	BMI Level	-0.319	[-0.810,	0.171]	0.443	**0.384**	**[0.184,**	**0.584]**	**0.001**
BMI Quadratic	Sex (women)	0.043	[-0.085,	0.172]	0.683	**0.135**	**[0.080,**	**0.189]**	**< 0.001**
	Cognitive function	-0.003	[-0.119,	0.113]	0.978	-0.009	[-0.054,	0.037]	0.742
	Sex x Cognitive function	-0.095	[-0.222,	0.032]	0.348	0.026	[-0.031,	0.084]	0.541
	Youth SED	-0.038	[-0.118,	0.043]	0.627	0.027	[-0.009,	0.062]	0.242
	Education	0.020	[-0.063,	0.104]	0.733	-0.015	[-0.051,	0.021]	0.563
	BMI Level	-0.002	[-0.166,	0.161]	0.978	-0.071	[-0.139,	-0.003]	0.083
	BMI Slope	-1.554	**[-1.810,**	**-1.298]**	**< 0.001**	**-1.006**	**[-1.146,**	**-0.866]**	**< 0.001**
χ2		83.966				137.465			
df		31				18			
CFI		0.992				0.991			
SRMR		0.015				0.011			

Models are latent growth curve models, with latent variables for BMI level, slope, and quadratic slope - see the path diagram in Figure 1. The same path diagram was used for both samples, except the US sample was modeled with adjustments for individual ages. All coeffecient values are multiple regression coefficients from the predictor onto the outcome, except the associations between the three BMI latent variables, which are covariances. P-values are corrected for multiple comparisons using the false discovery rate. SED = socioeconomic disadvantage.

For peer review only - http://bmjopen.bmj.com/site/about/guidelines.xhtml

For peer review only

For peer review only - http://bmjopen.bmj.com/site/about/guidelines.xhtml

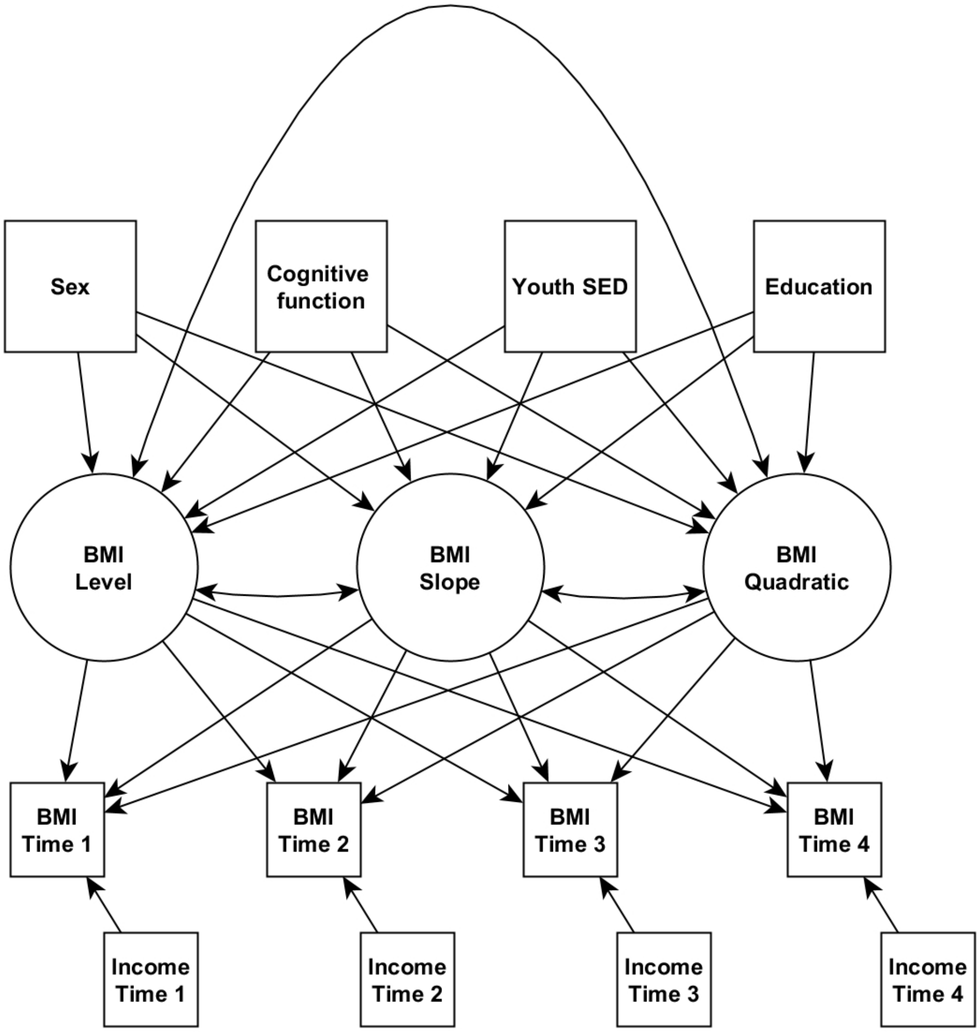

Figure 1. Path diagram of latent growth curve models used in primary analyses. Square boxes are observed variables, circles are latent variables. Single headed arrows are regressions or latent variable loadings, double headed arrows are covariances.

376x395mm (72 x 72 DPI)

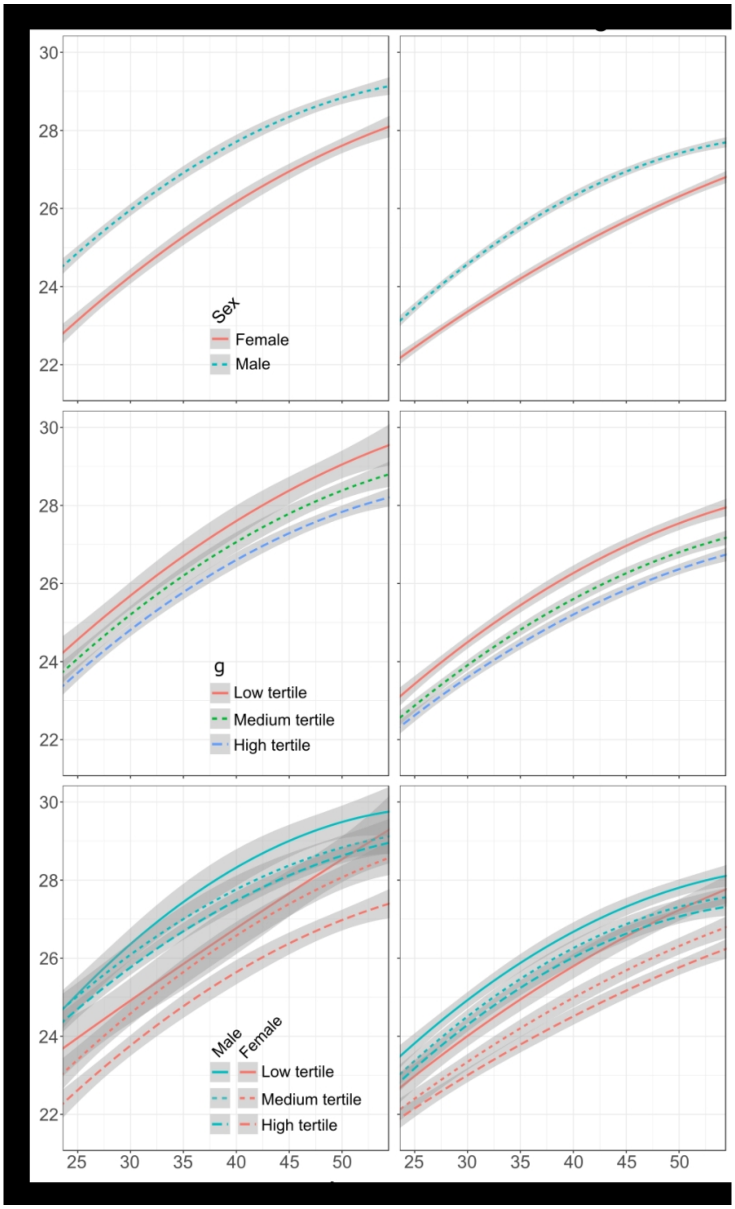

Figure 2. Quadratic growth curves of BMI, United States on the left, United Kingdom on the right, fitted to tertiles of cognitive function, and across different sexes. The top panels illustrates BMI between sexes, the middle panels illustrates BMI in different general cognitive function (g) tertiles, and the bottom panels illustrate the interaction between cognitive function tertiles and sex. Shaded areas represent the 95% error regions.

Supplementary Information for

How early-life cognitive and sociodemographic factors relate to the development of overweight and obesity in the UK and the US: a prospective cross-cohort study of the National Child Development Study and National Longitudinal Study of Youth 1979

For peer review only

This file includes:

Appendix 1 – Methods – Variables
Appendix 2 – Methods – Missing Data
Supplementary Tables S1 to S3

For peer review only - http://bmjopen.bmj.com/site/about/guidelines.xhtml

Appendix 1

Methods - Variables

BMI

BMI was calculated as weight divided by height^2, and where measurements were taken in Imperial units, they were converted to metric. Values of BMI greater than 70 or less than 12 were treated as biologically implausible [1] and the observations were removed from the dataset. From the NCDS, 0 such cases needed to be removed at age 23, 65 at age 33, 11 at age 42, and 4 at age 55. From the NLSY, only two such cases needed to be removed, at the 1985 wave.

General cognitive function

In the NLSY, cognitive function was assessed using the Armed Forces Qualification Test (AFQT), 1989 re-normed version. The AFQT consisted of four subtests assessing arithmetic reasoning, mathematical knowledge, word knowledge, and paragraph comprehension. The test was given in 1980, when participants were between 15 and 22 years old. The AFQT is a valid and reliable measure of general cognitive function, having been associated with outcomes including academic achievement and job performance [2,3]. To be consistent with earlier work [4,5], including *The Bell Curve* [6], we used the z-scored AFQT percentile score in our analyses.

In the NCDS, cognitive function was assessed using a general ability test, given to participants at age 11 [7]. The test consisted of 40 verbal and 40 nonverbal items, and the total score was z-scored for analysis. The reliability is high (test-retest Cronbach's α =

For peer review only - http://bmjopen.bmj.com/site/about/guidelines.xhtml

0.94), and the test is valid, as indicated by its high correlation (r = 0.69 to 0.93) with tests used for secondary school selection [8].

Youth SED

Due to historic data collection limitation, youth SED was composed differently in each sample, but both measures have been previously validated. In the US, youth SES was the sum of z-scored variables for parental income, education, and occupation status. Like AFQT scores, this variable has been previously used and validated in previous work [4 6]. In the UK, youth SED was the sum of six z-scored variables: father's social class at birth, father's social class at age 7, age at which the father left education, age at which the mother left education, parental housing tenure in childhood, and the number of people sharing a room in the household at age 7. This variable was composed to be consistent with earlier work [9].

Education

Education was measured in age at which an individual left school in the NCDS, and highest grade achieved in the NLSY, which was only converted to age at which an individual left school for descriptive purposes in Table 1. Each variable was independently z-scored for use in our models.

Income

Net family income was used in both samples. Net family income was derived from a comprehensive set of income questions in both samples. The possible sources came from

For peer review only - http://bmjopen.bmj.com/site/about/guidelines.xhtml

all earning members of the household, and included military income, wages, salaries, tips, unemployment compensation, child support, alimony, food stamps, welfare and disability benefits, interest and dividends, and others. The derivation of these variables in the NLSY is documented on the National Longitudinal Surveys website (https://www.nlsinfo.org/content/cohorts/nlsy79/topical-guide/income/income/) and in the NCDS the derivation of these variables was managed by the Centre for Longitudinal Studies [10]. Both income variables were top coded to ensure confidentiality of each sample's top earners, which also protects against some of the effects of large outlying observations.

Income variables were taken from the same wave as BMI variables with one exception. In the NCDS, income data were collected using a different instrument at age 55, the most recent available time point for BMI data. The age 55 measurement was missing more data than the age 50 measurement (652 more cases), and in addition to there being data collection inconsistencies at age 55, the variable had some problematic properties, e.g. smaller mean and median income statistics compared to those at age 50. Considering that model fit was sub-par using the age 55 measure, but much better using the age 50 measure, we used the age 50 income measure in all of the analyses we present.

For peer review only - http://bmjopen.bmj.com/site/about/guidelines.xhtml

Appendix 2

Methods - Missing Data

Study attrition and occasional non-response were present in both samples (see Table 1), though one of the benefits of our approach was that we could grapple with missing data with Full Information Maximum Likelihood estimation using observed information [11]. Standard errors were calculated with first order derivatives and *P*-values were calculated with a χ^2 test, both of which are procedures that can handle missing data.

Prior studies have used multiple imputation to test the robustness of analyses using some of these data [12 13]. They found the same results when comparing imputed and non-imputed model results.

For peer review only - http://bmjopen.bmj.com/site/about/guidelines.xhtml

Table S1. Full table of parameter estimates from each latent growth curve model of BMI growth in the US and UK.

United States				United Kingdom			
LHS	RHS	Estimate	SE	LHS	RHS	Estimate	SE
Latent Variables							
BMI Level	BMI ('85)	1		BMI Level	BMI (age 23)	1	
BMI Level	BMI ('94)	1		BMI Level	BMI (age 33)	1	
BMI Level	BMI ('06)	1		BMI Level	BMI (age 42)	1	
BMI Level	BMI ('14)	1		BMI Level	BMI (age 55)	1	
BMI Slope	BMI ('85)	0		BMI Slope	BMI (age 23)	0	
BMI Slope	BMI ('94)	0.9		BMI Slope	BMI (age 33)	1	
BMI Slope	BMI ('06)	2.1		BMI Slope	BMI (age 42)	1.9	
BMI Slope	BMI ('14)	2.9		BMI Slope	BMI (age 55)	3.2	
BMI Quadratic	BMI ('85)	0		BMI Quadratic	BMI (age 23)	0	
BMI Quadratic	BMI ('94)	0.81		BMI Quadratic	BMI (age 33)	1	
BMI Quadratic	BMI ('06)	4.41		BMI Quadratic	BMI (age 42)	3.61	
BMI Quadratic	BMI ('14)	8.41		BMI Quadratic	BMI (age 55)	10.24	
Regressions							
BMI Level	Sex (women)	-1.444	0.192	BMI Level	Sex (women)	-0.849	0.085
BMI Level	Cognitive function	0.105	0.166	BMI Level	Cognitive function	-0.196	0.068
BMI Level	Sex x Cognitive function	-0.525	0.192	BMI Level	Sex x Cognitive function	-0.053	0.086
BMI Level	Age ('79)	-0.320	1.399				
BMI Level	Youth SED	0.178	0.115	BMI Level	Youth SED	0.332	0.052
BMI Level	Education	-0.248	0.127	BMI Level	Education	-0.153	0.056
BMI Slope	Sex (women)	0.157	0.198	BMI Slope	Sex (women)	-0.327	0.090
BMI Slope	Cognitive function	0.092	0.177	BMI Slope	Cognitive function	0.036	0.075
BMI Slope	Sex x Cognitive function	0.295	0.195	BMI Slope	Sex x Cognitive function	-0.140	0.093
BMI Slope	Age ('79)	1.754	1.725				

For peer review only - http://bmjopen.bmj.com/site/about/guidelines.xhtml

BMI Slope	Youth SED	0.281	0.121	BMI Slope	Youth SED	0.028	0.057
BMI Slope	Education	-0.082	0.128	BMI Slope	Education	0.030	0.057
BMI Quadratic	Sex (women)	0.043	0.066	BMI Quadratic	Sex (women)	0.135	0.028
BMI Quadratic	Cognitive function	-0.003	0.059	BMI Quadratic	Cognitive function	-0.009	0.023
BMI Quadratic	Sex x Cognitive function	-0.095	0.065	BMI Quadratic	Sex x Cognitive function	0.026	0.029
BMI Quadratic	Age ('79)	-0.610	0.570				
BMI Quadratic	Youth SED	-0.038	0.041	BMI Quadratic	Youth SED	0.027	0.018
BMI Quadratic	Education	0.020	0.043	BMI Quadratic	Education	-0.015	0.018
BMI ('85)	Age ('85)	1.717	1.358				
BMI ('85)	Income ('85)	-0.098	0.110	BMI (age 23)	Income (age 23)	0.574	0.094
BMI ('94)	Age ('94)	-0.146	1.107				
BMI ('94)	Income ('94)	0.004	0.101	BMI (age 33)	Income (age 33)	-0.185	0.088
BMI ('06)	Age ('06)	-0.664	1.100				
BMI ('06)	Income ('06)	-0.076	0.076	BMI (age 42)	Income (age 42)	0.015	0.047
BMI ('14)	Age ('14)	-0.324	1.584				
BMI ('14)	Income ('14)	-0.142	0.080	BMI (age 55)	Income (age 50)	0.071	0.058
Variances and covariances							
BMI ('85)	BMI ('85)	0.010	0.000	BMI (age 23)	BMI (age 23)	0.010	#N/A
BMI ('94)	BMI ('94)	4.759	0.156	BMI (age 33)	BMI (age 33)	7.578	0.063
BMI ('06)	BMI ('06)	3.603	0.183	BMI (age 42)	BMI (age 42)	2.364	0.087
BMI ('14)	BMI ('14)	5.745	0.514	BMI (age 55)	BMI (age 55)	5.762	0.677
BMI Level	BMI Level	15.309	0.371	BMI Level	BMI Level	9.064	0.124
BMI Slope	BMI Slope	7.299	0.359	BMI Slope	BMI Slope	5.183	0.186
BMI Quadratic	BMI Quadratic	0.436	0.057	BMI Quadratic	BMI Quadratic	0.255	0.034
BMI Level	BMI Slope	-0.319	0.250	BMI Level	BMI Slope	0.384	0.102
BMI Level	BMI Quadratic	-0.002	0.083	BMI Level	BMI Quadratic	-0.071	0.035
BMI Slope	BMI Quadratic	-1.554	0.131	BMI Slope	BMI Quadratic	-1.006	0.071
Sex (women)	Sex (women)	0.250	0.000	Sex (women)	Sex (women)	0.249	0.000

For peer review only

For peer review only - http://bmjopen.bmj.com/site/about/guidelines.xhtml

Sex (women)	Cognitive function	-0.041	0.000	Sex (women)	Cognitive function	0.031	0.000
Sex (women)	Sex x Cognitive function	0.052	0.000	Sex (women)	Sex x Cognitive function	0.064	0.000
Sex (women)	Age ('79)	0.003	0.000				
Sex (women)	Youth SED	0.020	0.000	Sex (women)	Youth SED	0.011	0.000
Sex (women)	Education	0.002	0.000	Sex (women)	Education	-0.011	0.000
Sex (women)	Age ('85)	0.004	0.000				
Sex (women)	Income ('85)	-0.017	0.000	Sex (women)	Income (age 23)	0.013	0.000
Sex (women)	Age ('94)	0.003	0.000				
Sex (women)	Income ('94)	-0.015	0.000	Sex (women)	Income (age 33)	0.004	0.000
Sex (women)	Age ('06)	0.004	0.000				
Sex (women)	Income ('06)	-0.045	0.000	Sex (women)	Income (age 42)	-0.025	0.000
Sex (women)	Age ('14)	0.003	0.000				
Sex (women)	Income ('14)	-0.094	0.000	Sex (women)	Income (age 50)	-0.053	0.000
Cognitive function	Cognitive function	0.800	0.000	Cognitive function	Cognitive function	0.862	0.000
Cognitive function	Sex x Cognitive function	0.344	0.000	Cognitive function	Sex x Cognitive function	0.442	0.000
Cognitive function	Age ('79)	0.031	0.000				
Cognitive function	Youth SED	-0.375	0.000	Cognitive function	Youth SED	-0.338	0.000
Cognitive function	Education	0.536	0.000	Cognitive function	Education	0.369	0.000
Cognitive function	Age ('85)	0.031	0.000				
Cognitive function	Income ('85)	0.073	0.000	Cognitive function	Income (age 23)	0.013	0.000
Cognitive function	Age ('94)	0.030	0.000				
Cognitive function	Income ('94)	0.228	0.000	Cognitive function	Income (age 33)	0.103	0.000
Cognitive function	Age ('06)	0.028	0.000				
Cognitive function	Income ('06)	0.353	0.000	Cognitive function	Income (age 42)	0.147	0.000
Cognitive function	Age ('14)	0.030	0.000				
Cognitive function	Income ('14)	0.483	0.000	Cognitive function	Income (age 50)	0.206	0.000
Sex x Cognitive function	Sex x Cognitive function	0.363	0.000	Sex x Cognitive function	Sex x Cognitive function	0.451	0.000
Sex x Cognitive function	Age ('79)	0.014	0.000				

For peer review only

Sex x Cognitive function	Youth SED	-0.163	0.000	Sex x Cognitive function	Youth SED	-0.185	0.000
Sex x Cognitive function	Education	0.236	0.000	Sex x Cognitive function	Education	0.176	0.000
Sex x Cognitive function	Age ('85)	0.013	0.000				
Sex x Cognitive function	Income ('85)	0.034	0.000	Sex x Cognitive function	Income (age 23)	0.017	0.000
Sex x Cognitive function	Age ('94)	0.014	0.000				
Sex x Cognitive function	Income ('94)	0.100	0.000	Sex x Cognitive function	Income (age 33)	0.050	0.000
Sex x Cognitive function	Age ('06)	0.012	0.000				
Sex x Cognitive function	Income ('06)	0.159	0.000	Sex x Cognitive function	Income (age 42)	0.070	0.000
Sex x Cognitive function	Age ('14)	0.013	0.000				
Sex x Cognitive function	Income ('14)	0.185	0.000	Sex x Cognitive function	Income (age 50)	0.089	0.000
Age ('79)	Age ('79)	0.049	0.000				
Age ('79)	Youth SED	-0.005	0.000				
Age ('79)	Education	-0.001	0.000				
Age ('79)	Age ('85)	0.049	0.000				
Age ('79)	Income ('85)	0.011	0.000				
Age ('79)	Age ('94)	0.049	0.000				
Age ('79)	Income ('94)	0.012	0.000				
Age ('79)	Age ('06)	0.049	0.000				
Age ('79)	Income ('06)	0.001	0.000				
Age ('79)	Age ('14)	0.048	0.000				
Age ('79)	Income ('14)	-0.004	0.000				
Youth SED	Youth SED	0.760	0.000	Youth SED	Youth SED	0.909	0.000
Youth SED	Education	-0.418	0.000	Youth SED	Education	-0.397	0.000
Youth SED	Age ('85)	-0.006	0.000				
Youth SED	Income ('85)	-0.082	0.000	Youth SED	Income (age 23)	0.012	0.000
Youth SED	Age ('94)	-0.006	0.000				
Youth SED	Income ('94)	-0.176	0.000	Youth SED	Income (age 33)	-0.080	0.000
Youth SED	Age ('06)	-0.004	0.000				

For peer review only - http://bmjopen.bmj.com/site/about/guidelines.xhtml

Youth SED	Income ('06)	-0.278	0.000	Youth SED	Income (age 42)	-0.151	0.000
Youth SED	Age ('14)	-0.006	0.000				
Youth SED	Income ('14)	-0.378	0.000	Youth SED	Income (age 50)	-0.194	0.000
Education	Education	0.937	0.000	Education	Education	0.982	0.000
Education	Age ('85)	0.000	0.000				
Education	Income ('85)	0.066	0.000	Education	Income (age 23)	-0.033	0.000
Education	Age ('94)	-0.001	0.000				
Education	Income ('94)	0.242	0.000	Education	Income (age 33)	0.100	0.000
Education	Age ('06)	-0.001	0.000				
Education	Income ('06)	0.400	0.000	Education	Income (age 42)	0.180	0.000
Education	Age ('14)	0.000	0.000				
Education	Income ('14)	0.552	0.000	Education	Income (age 50)	0.249	0.000
Age ('85)	Age ('85)	0.050	0.000				
Age ('85)	Income ('85)	0.011	0.000				
Age ('85)	Age ('94)	0.048	0.000				
Age ('85)	Income ('94)	0.013	0.000				
Age ('85)	Age ('06)	0.049	0.000				
Age ('85)	Income ('06)	0.002	0.000				
Age ('85)	Age ('14)	0.048	0.000				
Age ('85)	Income ('14)	-0.003	0.000				
Income ('85)	Income ('85)	0.269	0.000	Income (age 23)	Income (age 23)	0.082	0.000
Income ('85)	Age ('94)	0.011	0.000				
Income ('85)	Income ('94)	0.101	0.000	Income (age 23)	Income (age 33)	0.024	0.000
Income ('85)	Age ('06)	0.011	0.000				
Income ('85)	Income ('06)	0.111	0.000	Income (age 23)	Income (age 42)	0.038	0.000
Income ('85)	Age ('14)	0.011	0.000				
Income ('85)	Income ('14)	0.140	0.000	Income (age 23)	Income (age 50)	0.034	0.000
Age ('94)	Age ('94)	0.050	0.000				

For peer review only

Age ('94)	Income ('94)	0.012	0.000				
Age ('94)	Age ('06)	0.048	0.000				
Age ('94)	Income ('06)	0.002	0.000				
Age ('94)	Age ('14)	0.048	0.000				
Age ('94)	Income ('14)	-0.004	0.000				
Income ('94)	Income ('94)	0.450	0.000	Income (age 33)	Income (age 33)	0.287	0.000
Income ('94)	Age ('06)	0.012	0.000				
Income ('94)	Income ('06)	0.388	0.000	Income (age 33)	Income (age 42)	0.155	0.000
Income ('94)	Age ('14)	0.012	0.000				
Income ('94)	Income ('14)	0.446	0.000	Income (age 33)	Income (age 50)	0.170	0.000
Age ('06)	Age ('06)	0.049	0.000				
Age ('06)	Income ('06)	0.001	0.000				
Age ('06)	Age ('14)	0.048	0.000				
Age ('06)	Income ('14)	-0.005	0.000				
Income ('06)	Income ('06)	1.045	0.000	Income (age 42)	Income (age 42)	0.628	0.000
Income ('06)	Age ('14)	0.001	0.000				
Income ('06)	Income ('14)	0.930	0.000	Income (age 42)	Income (age 50)	0.343	0.000
Age ('14)	Age ('14)	0.049	0.000				
Age ('14)	Income ('14)	-0.002	0.000				
Income ('14)	Income ('14)	1.643	0.000	Income (age 50)	Income (age 50)	0.937	0.000
Intercepts							
BMI ('85)		0.000	0.000	BMI (age 23)		0.000	0.000
BMI ('94)		0.000	0.000	BMI (age 33)		0.000	0.000
BMI ('06)		0.000	0.000	BMI (age 42)		0.000	0.000
BMI ('14)		0.000	0.000	BMI (age 55)		0.000	0.000
Sex (women)		0.511	0.000	Sex (women)		0.530	0.000
Cognitive function		0.287	0.000	Cognitive function		0.199	0.000
Sex x Cognitive function		0.106	0.000	Sex x Cognitive function		0.136	0.000

For peer review only - http://bmjopen.bmj.com/site/about/guidelines.xhtml

Age ('79)	1.807	0.000			
Youth SED	-0.206	0.000	Youth SED	-0.087	0.000
Education	0.303	0.000	Education	0.041	0.000
Age ('85)	2.353	0.000			
Income ('85)	-0.552	0.000	Income (age 23)	-0.961	0.000
Age ('94)	3.297	0.000			
Income ('94)	-0.039	0.000	Income (age 33)	-0.199	0.000
Age ('06)	4.467	0.000			
Income ('06)	0.541	0.000	Income (age 42)	0.349	0.000
Age ('14)	5.347	0.000			
Income ('14)	0.688	0.000	Income (age 50)	0.717	0.000
BMI Level	20.979	1.095	BMI Level	23.645	0.116
BMI Slope	6.103	2.833	BMI Slope	1.929	0.119
BMI Quadratic	-0.822	1.320	BMI Quadratic	-0.186	0.032

LHS = left-hand side; RHS = right-hand side; SED = socioeconomic disadvantage.

For peer review only

For peer review only - http://bmjopen.bmj.com/site/about/guidelines.xhtml

Table S2. Coefficient estimates from models of BMI growth, cognitive function, and sociodemographic variables, income excluded.

		United States				United Kingdom			
Outcome	Predictor	Estimate	95%	C.I.	*P*	Estimate	95%	C.I.	*P*
BMI Level	Sex (women)	**-1.572**	**[-1.868,**	**-1.276]**	**< 0.001**	**-0.840**	**[-0.979,**	**-0.701]**	**< 0.001**
	Cognitive function	0.123	[-0.141,	0.387]	0.543	**-0.195**	**[-0.301,**	**-0.089]**	**0.001**
	Sex x Cognitive function	**-0.394**	**[-0.691,**	**-0.097]**	**0.033**	-0.014	[-0.152,	0.123]	0.840
	Youth SED	0.182	[-0.003,	0.367]	0.122	**0.277**	**[0.192,**	**0.363]**	**< 0.001**
	Education	**-0.339**	**[-0.534,**	**-0.143]**	**0.004**	**-0.206**	**[-0.303,**	**-0.110]**	**< 0.001**
BMI Slope	Sex (women)	0.148	[-0.163,	0.459]	0.543	**-0.391**	**[-0.542,**	**-0.239]**	**< 0.001**
	Cognitive function	0.019	[-0.258,	0.296]	0.893	-0.023	[-0.142,	0.096]	0.743
	Sex x Cognitive function	0.257	[-0.049,	0.563]	0.180	-0.118	[-0.270,	0.034]	0.231
	Youth SED	0.209	[0.018,	0.401]	0.096	0.025	[-0.071,	0.122]	0.683
	Education	-0.069	[-0.265,	0.127]	0.627	0.065	[-0.027,	0.157]	0.272
	BMI Level	0.416	[0.012,	0.819]	0.112	0.399	[-0.206,	1.003]	0.294
BMI Quadratic	Sex (women)	0.043	[-0.059,	0.144]	0.567	**0.146**	**[0.098,**	**0.194]**	**< 0.001**
	Cognitive function	0.009	[-0.081,	0.099]	0.893	0.012	[-0.026,	0.050]	0.629
	Sex x Cognitive function	-0.085	[-0.185,	0.016]	0.180	0.020	[-0.030,	0.069]	0.558
	Youth SED	-0.013	[-0.078,	0.052]	0.789	0.027	[-0.004,	0.058]	0.183
	Education	0.014	[-0.059,	0.078]	0.789	-0.025	[-0.053,	0.004]	0.183
	BMI Level	**-0.203**	**[-0.336,**	**-0.071]**	**0.012**	-0.072	[-0.211,	0.067]	0.430
	BMI Slope	**-1.501**	**[-1.724,**	**-1.279]**	**< 0.001**	**-1.041**	**[-1.213,**	**-0.870]**	**< 0.001**
χ2		59.740				254.238			
df		19				6			
CFI		0.996				0.985			
SRMR		0.006				0.015			

Models are latent growth curve models, with latent variables for BMI level, slope, and quadratic slope. The same path diagram was used for both samples, except the US sample was modelled with adjustments for individual ages. All coefficient values are multiple regression coefficients from

For peer review only - http://bmjopen.bmj.com/site/about/guidelines.xhtml

the predictor onto the outcome, except the associations between the three BMI latent variables, which are covariances. P-values are corrected for multiple comparisons using the false discovery rate.

For peer review only

For peer review only - http://bmjopen.bmj.com/site/about/guidelines.xhtml

Table S3. Coefficient estimates from models of BMI growth, cognitive function, and sociodemographic variables, income and education excluded.

		United States				United Kingdom			
Outcome	Predictor	Estimate	95%	C.I.	*P*	Estimate	95%	C.I.	*P*
BMI Level	Sex (women)	**-1.628**	**[-1.922,**	**-1.333]**	**< 0.001**	**-0.926**	**[-1.049,**	**-0.803]**	**< 0.001**
	Cognitive function	-0.060	[-0.306,	0.187]	0.732	**-0.231**	**[-0.321,**	**-0.141]**	**< 0.001**
	Sex x Cognitive function	**-0.400**	**[-0.697,**	**-0.103]**	**0.025**	-0.019	[-0.139,	0.101]	0.810
	Youth SED	**0.274**	**[0.101,**	**0.448]**	**0.010**	**0.343**	**[0.270,**	**0.415]**	**< 0.001**
BMI Slope	Sex (women)	0.146	[-0.163,	0.455]	0.534	**-0.401**	**[-0.546,**	**-0.255]**	**< 0.001**
	Cognitive function	-0.022	[-0.282,	0.238]	0.870	0.026	[-0.085,	0.138]	0.793
	Sex x Cognitive function	0.265	[-0.040,	0.570]	0.151	-0.148	[-0.293,	-0.004]	0.067
	Youth SED	**0.231**	**[0.051,**	**0.412]**	**0.030**	0.018	[-0.068,	0.103]	0.793
	BMI Level	0.386	[-0.017,	0.789]	0.129	**0.330**	**[0.170,**	**0.490]**	**< 0.001**
BMI Quadratic	Sex (women)	0.042	[-0.059,	0.143]	0.565	**0.149**	**[0.102,**	**0.196]**	**< 0.001**
	Cognitive function	0.017	[-0.068,	0.102]	0.749	-0.004	[-0.040,	0.033]	0.849
	Sex x Cognitive function	-0.086	[-0.186,	0.014]	0.151	0.029	[-0.019,	0.077]	0.324
	Youth SED	-0.017	[-0.078,	0.044]	0.732	**0.032**	**[0.004,**	**0.060]**	**0.045**
	BMI Level	**-0.193**	**[-0.326,**	**-0.061]**	**0.016**	-0.058	[-0.114,	-0.001]	0.067
	BMI Slope	**-1.497**	**[-1.718,**	**-1.275]**	**< 0.001**	**-1.076**	**[-1.188,**	**-0.964]**	**< 0.001**
χ2		58.438				269.375			
df		18				5			
CFI		0.996				0.985			
SRMR		0.006				0.016			

Models are latent growth curve models, with latent variables for BMI level, slope, and quadratic slope. The same path diagram was used for both samples, except the US sample was modeled with adjustments for individual ages. All coeffecient values are multiple regression coefficients from the predictor onto the outcome, except the associations between the three BMI latent variables, which are covariances. P-values are corrected for multiple comparisons using the false discovery rate.

For peer review only - http://bmjopen.bmj.com/site/about/guidelines.xhtml

For peer review only

1. Li W, Kelsey JL, Zhang Z, et al. Small-area estimation and prioritizing communities for obesity control in Massachusetts. *Am J Public Health* 2009;99(3):511-19.
2. Palmer P, Hartke DD, Ree MJ, et al. Armed Services Vocational Aptitude Battery (ASVAB): Alternate Forms Reliability (Forms 8, 9, 10, and 11): AIR FORCE HUMAN RESOURCES LAB BROOKS AFB TX, 1988.
3. Welsh Jr JR, Kucinkas SK, Curran LT. Armed services vocational battery (ASVAB): Integrative review of validity studies: OPERATIONAL TECHNOLOGIES CORP SAN ANTONIO TX, 1990.
4. Der G, Batty GD, Deary IJ. The association between IQ in adolescence and a range of health outcomes at 40 in the 1979 US National Longitudinal Study of Youth. *Intelligence* 2009;37(6):573-80.
5. Wraw C, Deary IJ, Der G, et al. Maternal and offspring intelligence in relation to BMI across childhood and adolescence. *Int J Obesity* 2018 doi: 10.1038/s41366-018-0009-1
6. Herrnstein RJ, Murray C. Bell curve: Intelligence and class structure in American life: Simon and Schuster 2010.
7. Pigeon D. Tests used in the 1954 and 1957 surveys. *The home and the school* 1964:129-32.
8. Douglas JWB. The home and the school: MacGibbon & Kee London 1964.
9. Bridger E, Daly M. Does cognitive ability buffer the link between childhood disadvantage and adult health? *Health Psychol* 2017;36(10):966.
10. Goodman A, Joyce R, Smith JP. The long shadow cast by childhood physical and mental problems on adult life. *Proceedings of the National Academy of Sciences* 2011:201016970.
11. Savalei V. Expected versus observed information in SEM with incomplete normal and nonnormal data. *Psychological methods* 2010;15(4):352.
12. Wraw C, Deary IJ, Gale CR, et al. Intelligence in youth and health at age 50. *Intelligence* 2015;53:23-32.
13. Wraw C, Deary IJ, Der G, et al. Intelligence in youth and mental health at age 50. *Intelligence* 2016;58:69-79.

For peer review only

Reporting checklist for cohort study.

Based on the STROBE cohort guidelines.

Instructions to authors

Complete this checklist by entering the page numbers from your manuscript where readers will find each of the items listed below.

Your article may not currently address all the items on the checklist. Please modify your text to include the missing information. If you are certain that an item does not apply, please write "n/a" and provide a short explanation.

Upload your completed checklist as an extra file when you submit to a journal.

In your methods section, say that you used the STROBE cohortreporting guidelines, and cite them as:

von Elm E, Altman DG, Egger M, Pocock SJ, Gotzsche PC, Vandenbroucke JP. The Strengthening the Reporting of Observational Studies in Epidemiology (STROBE) Statement: guidelines for reporting observational studies.

For peer review only

		Reporting Item	Page Number
**Title and abstract**			
Title	#1a	Indicate the study's design with a commonly used term in the title or the abstract	1
Abstract	#1b	Provide in the abstract an informative and balanced summary of what was done and what was found	2-3

**Introduction**			
Background / rationale	#2	Explain the scientific background and rationale for the investigation being reported	5-6
Objectives	#3	State specific objectives, including any prespecified hypotheses	6-7
**Methods**			
Study design	#4	Present key elements of study design early in the paper	
Setting	#5	Describe the setting, locations, and relevant dates, including periods of recruitment, exposure, follow-up, and data collection	7-8
Eligibility criteria	#6a	Give the eligibility criteria, and the sources and methods of selection of participants. Describe methods of follow-up.	7-9
Eligibility criteria	#6b	For matched studies, give matching criteria and number of exposed and unexposed	N/A-No matching
Variables	#7	Clearly define all outcomes, exposures, predictors, potential confounders, and effect modifiers. Give diagnostic criteria, if applicable	8
Data sources / measurement	#8	For each variable of interest give sources of data and details of methods of assessment (measurement). Describe comparability of assessment methods if there is more than one group. Give information separately for	8

For peer review only

		for exposed and unexposed groups if applicable.	
Bias	#9	Describe any efforts to address potential sources of bias	8-9
Study size	#10	Explain how the study size was arrived at	7-9
Quantitative variables	#11	Explain how quantitative variables were handled in the analyses. If applicable, describe which groupings were chosen, and why	8-9
Statistical methods	#12a	Describe all statistical methods, including those used to control for confounding	8-9
Statistical methods	#12b	Describe any methods used to examine subgroups and interactions	8-9
Statistical methods	#12c	Explain how missing data were addressed	8-9
Statistical methods	#12d	If applicable, explain how loss to follow-up was addressed	8-9
Statistical methods	#12e	Describe any sensitivity analyses	12-13
**Results**			
Participants	#13a	Report numbers of individuals at each stage of study—eg numbers potentially eligible, examined for eligibility, confirmed eligible, included in the study, completing follow-up, and analysed. Give information separately for for exposed and unexposed groups if applicable.	Table 1

For peer review only

Participants	#13b	Give reasons for non-participation at each stage	N/A-cohort study
Participants	#13c	Consider use of a flow diagram	Cf. Figure 1
Descriptive data	#14a	Give characteristics of study participants (eg demographic, clinical, social) and information on exposures and potential confounders. Give information separately for exposed and unexposed groups if applicable.	Table 1
Descriptive data	#14b	Indicate number of participants with missing data for each variable of interest	Table 1
Descriptive data	#14c	Summarise follow-up time (eg, average and total amount)	9-10, Table 1
Outcome data	#15	Report numbers of outcome events or summary measures over time. Give information separately for exposed and unexposed groups if applicable.	Table 1
Main results	#16a	Give unadjusted estimates and, if applicable, confounder-adjusted estimates and their precision (eg, 95% confidence interval). Make clear which confounders were adjusted for and why they were included	11-12
Main results	#16b	Report category boundaries when continuous variables were categorized	N/A-no variables were made into categories

For peer review only

Main results	#16c	If relevant, consider translating estimates of relative risk into absolute risk for a meaningful time period	N/A-risk not a factor
Other analyses	#17	Report other analyses done—e.g., analyses of subgroups and interactions, and sensitivity analyses	12-13
**Discussion**			
Key results	#18	Summarise key results with reference to study objectives	13-15
Limitations	#19	Discuss limitations of the study, taking into account sources of potential bias or imprecision. Discuss both direction and magnitude of any potential bias.	15-16
Interpretation	#20	Give a cautious overall interpretation considering objectives, limitations, multiplicity of analyses, results from similar studies, and other relevant evidence.	16-17
Generalisability	#21	Discuss the generalisability (external validity) of the study results	15-17
**Other Information**			
Funding	#22	Give the source of funding and the role of the funders for the present study and, if applicable, for the original study on which the present article is based	18

None The STROBE checklist is distributed under the terms of the Creative Commons Attribution License CC-BY. This checklist can be completed online using https://www.goodreports.org/, a tool made by the EQUATOR Network in collaboration with Penelope.ai

For peer review only

BMJ Open

How youth cognitive and sociodemographic factors relate to the development of overweight and obesity in the UK and the US: a prospective cross-cohort study of the National Child Development Study and National Longitudinal Study of Youth 1979

Journal:	*BMJ Open*
Manuscript ID	bmjopen-2019-033011.R1
Article Type:	Original research
Date Submitted by the Author:	21-Oct-2019
Complete List of Authors:	Altschul, Drew; The University of Edinburgh, Psychology<br>Wraw, Christina; National Health Service Scotland<br>Gale, Catharine; MRC Epidemiology Resource Centre<br>Deary, Ian; University of Edinburgh, Department of Psychology
**Primary Subject Heading**:	Epidemiology
Secondary Subject Heading:	Public health, Nutrition and metabolism, Mental health, Health economics, Global health
Keywords:	obesity, overweight, longitudinal studies, cognition, socioeconomic factors

SCHOLARONE™
Manuscripts

How youth cognitive and sociodemographic factors relate to the development of overweight and obesity in the UK and the US: a prospective cross-cohort study of the National Child Development Study and National Longitudinal Study of Youth 1979

Short title: Cognitive and sociodemographic factors and obesity

Drew M Altschul[a,b,c*], Christina Wraw[c,d], Catharine R Gale,[a,c,e] Ian J Deary[a,c]

[a] Department of Psychology, The University of Edinburgh, Edinburgh, EH8 9JZ, UK.

[b] Mental Health Data Science Scotland, Edinburgh, EH8 9JZ, UK.

[c] Centre for Cognitive Ageing and Cognitive Epidemiology, Department of Psychology, University of Edinburgh, Edinburgh, EH8 9JZ, UK.

[d] National Health Service Scotland, Edinburgh, EH12 9EB, UK

[e] MRC Lifecourse Epidemiology Unit, University of Southampton, Southampton, SO16 6YD, UK.

*Correspondence to: dmaltschul@gmail.com; 7 George Square, Edinburgh, UK, EH8 9JZ; +44 0131 650 4007

Key words: cognition, socioeconomic factors, obesity, overweight, longitudinal studies

Word count: 4338

For peer review only - http://bmjopen.bmj.com/site/about/guidelines.xhtml

ABSTRACT

Objectives

We investigated how early-life cognitive and sociodemographic factors are associated with the etiology of overweight and obesity. We examined both onset (who is at early risk for overweight and obesity), and development (who gains weight and when).

Design

Prospective cohort study.

Setting

We used data from the United States National Longitudinal Study of Youth 1979 (NLSY) and the United Kingdom National Child Development Study (NCDS); most of both studies completed a cognitive function test in youth.

Participants

12,686 and 18,558 members of the NLSY and NCDS, respectively, with data on validated measures of youth cognitive function, youth socioeconomic disadvantage (e.g. parental occupational class and time spent in school), and educational attainment. Height, weight, and income data were available from across adulthood, from individuals' 20s into their 50s.

Primary and secondary outcome measures

Body Mass Index (BMI) for four time points in adulthood. We modeled gain in BMI using latent growth curve models to capture linear and quadratic components of change in BMI over time.

Results

For peer review only - http://bmjopen.bmj.com/site/about/guidelines.xhtml

For peer review only

Across cohorts, higher cognitive function was associated with lower overall BMI. In the UK, 1 standard deviation higher score in cognitive function was associated with lower BMI (β=-0.20, 95%CI:-0.33 – -0.06 kg/m²). In Americans, this was true only for women (β=-0.53, 95%CI:-0.90 – -0.15 kg/m²), for whom higher cognitive function was associated with lower BMI. In British participants only, we found limited evidence for negative and positive associations, respectively, between education (β=-0.15, 95%CI:-0.26 – -0.04 kg/m²) and socioeconomic disadvantage (β=0.33, 95%CI:0.23 – 0.43 kg/m²) and higher BMI. Overall, no cognitive or socioeconomic factors in youth were associated with longitudinal changes in BMI.

Conclusions

While sociodemographic and particularly cognitive factors can explain some patterns in individuals' overall weight levels, differences in who gains weight in adulthood could not be explained by any of these factors.

For peer review only - http://bmjopen.bmj.com/site/about/guidelines.xhtml

STRENGTHS AND LIMITATIONS OF THIS STUDY

- This study used two independent cohorts from the US and UK, each with more than 30 years of follow-up, allowing us to model levels and trajectories of BMI cross-culturally.
- The cohorts are representative of the eras and nations they developed in, so our findings are limited to middle-aged Caucasian populations in Western nations.
- Cognitive and sociodemographic variables were available in both samples, and allowed us to compare the associations of these factors with the development and progression of overweight and obesity.
- More work is required to clarify if and how cognitive and sociodemographic factors are linked to overweight and obesity across the life course.

For peer review only

INTRODUCTION

Obesity is increasingly prevalent worldwide,[1] doubling between 1980 and 2015 in 73 countries, among both children and adults.[2] Obesity and overweight is dangerously prevalent in both the US and UK;[2] in 2015 more than 196,000 deaths in the US and 26,000 deaths in the UK were attributable to overweight and obesity. Furthermore, obesity and overweight are linked to a range of physical ailments, including cardiovascular disease[3], cancer,[4] type II diabetes and osteoarthritis,[5] as well as mental illnesses, such as depression,[6,7] anxiety,[8] and bipolar disorder.[9] It is thus an important issue in public health to identify early life factors that are associated with obesity and overweight in adulthood. To this end, we investigated the relationships of sociodemographics and general cognitive function in youth with the onset and growth of obesity and overweight from the beginning of adulthood into middle age.

From the beginning of life, inherent biological factors such as sex play a role in determining bodily health,[10] quantified through measures like body mass index (BMI), which is used to define who is overweight and obese. BMI is estimated at between 40% and 70% heritable.[11] On the other hand, early life socioeconomic disadvantage (SED) and adverse experiences are associated with higher risk of overweight and obesity,[12,13] though these findings are confounded by ethnic and geographic disparities.[14,15]

Cognitive function is also an important predictor of both physical and mental health outcomes,[16-21] and lower cognitive function is linked with obesity later in life.[22-24] Youth cognitive function in particular is the gold standard for cognitive epidemiological studies that seek to investigate the long-term associations between cognitive function and the development of disease; a measure of cognitive function from youth limits confounding. Cognitive function declines on average with age[25] and in the presence of disease comorbidity.[26-28] Moreover, obesity itself has been linked to high incidence of Alzheimer's,[29]

For peer review only

For peer review only - http://bmjopen.bmj.com/site/about/guidelines.xhtml

Parkinson's,[30] and other sources of cognitive decline and dementia in older age.[31] Therefore, early-life cognitive function measures are especially important for understanding the relationships between cognitive function and overweight and obesity.

Gradients in SED affect women's physical health differently from men's, such that greater SED in women is more consistently associated with poorer health.[32 33] Therefore, might gradients in cognitive function also affect women's health similarly, i.e. with stronger associations between cognitive function and health than in men? In 1996, obesity was more likely in English women who were more disadvantaged, e.g. had lower occupational status, but no such association was present in English men.[32] In related health conditions such as diabetes,[33] heart disease,[34 35] and hypertension,[36] women coming from greater disadvantage also had poorer disease outcomes. However, associations between early-life SED and health might be at least partially confounded by differential effects of early life cognitive function, which are also associated with later life health outcomes, including overweight and obesity.[37] Cognitive function is an important predictor of later life health in both sexes, but it plays a stronger role in women. Evidence of this has been found in both the US[38] and UK,[35 37] for high blood pressure, stroke, and coronary heart disease.

Considerable longitudinal research is carried out in the US and UK, but not nearly as much research has examined whether major correlates of illness are comparable between these nations. Our main goal in the present study was thus to compare, between both countries, whether cognitive function or SED in youth are associated with BMI from young adulthood to middle age. In particular, we wished to investigate the role of general cognitive function and its potential interaction with sex, in BMI changes from early adulthood to middle age. To this end, we analyzed two large, comparable, longitudinal samples from the US and UK, which allowed us to model growth curves and determine which early-life

For peer review only - http://bmjopen.bmj.com/site/about/guidelines.xhtml

cognitive and sociodemographic factors are associated with higher BMI over the course of adulthood.

METHODS

The American and British Samples are, respectively, the National Longitudinal Study of Youth 1979 (NLSY) and the National Child Development Study (NCDS). Both studies have followed participants from youth, when a test of general cognitive function was given. Sociodemographic variables from youth were also available, allowing us to consider other known factors that contribute to overweight and obesity. BMI was tracked longitudinally from participants' 20s into their 50s, Specific details on the individual samples follow.

The National Longitudinal Study of Youth 1979 (NLSY)

The NLSY was initially sampled from non-institutionalized young Americans born between 1956 and 1964; aged 14 – 21 years and living in the US at the beginning of 1979. The original sample consisted of 12,686 participants, and was ethnically representative of the US at that time; 16% of participants were "Hispanic", 25% were "black", and 59% were "non-Black non-Hispanic"; a total of 7506 individuals were in this last category, so 5172 NLSY participants were either Black or Hispanic. The initial interview took place in 1979, and respondents were regularly re-interviewed up to 2014, which is when the most recent data were available. 3396 individuals participated in giving height and weight measurements in 2014 (Table 1). The ethnic categories were less specific than in the NCDS, so for example, some Asian-Americans were likely included as "non-Black non-Hispanic", though in 1980 only 1.5% of the US population was of Asian or Pacific Islander descent.

For peer review only - http://bmjopen.bmj.com/site/about/guidelines.xhtml

The National Child Development Study (NCDS)

Participants of the NCDS were born in the UK during one week in March 1958. As originally intended, the NCDS was designed to study stillbirth and death in infancy. Later developments in the survey have resulted in multiple follow-ups at regular intervals. Eleven waves of data have been collected, the first at birth and the most recent in 2013, when participants were 55 years old. Of the initial 18,558 participants, 12,440 (67%) were "Euro-Caucasian". By the 2013 wave, 6861 (37%) individuals participated in giving height and weight measurements, again see Table 1. Analyses were limited to only non-minority ethnic group individuals to allow between-country comparisons.[39 40]

Exposures

In the NLSY, general cognitive function was assessed using the Armed Forces Qualification Test (AFQT), 1989 re-normed version. The AFQT consisted of four subtests assessing arithmetic reasoning, mathematical knowledge, word knowledge, and paragraph comprehension. The test was given in 1980, when participants were between 15 and 22 years old. The AFQT is a valid and reliable measure of general cognitive function, having been associated with outcomes including academic achievement and job performance [41 42]. To be consistent with earlier work [20 43], including *The Bell Curve* [44], we used the z-scored AFQT percentile score in our analyses. In the NCDS, cognitive function was assessed using a general ability test, given to participants at age 11 [45]. The test consisted of 40 verbal and 40 nonverbal items, and the total score was z-scored for analysis. The reliability is high (test-retest Cronbach's $\alpha = 0.94$), and the test is valid, as indicated by its high correlation ($r = 0.69$ to 0.93) with tests used for secondary school selection [46].

For peer review only - http://bmjopen.bmj.com/site/about/guidelines.xhtml

Due to historic data collection limitation, youth SED was composed differently in each sample, but both measures have been previously validated. In the US, youth SES was the sum of z-scored variables for parental income, education, and occupation status. Like AFQT scores, this variable has been previously used and validated in previous work [43 44]. In the UK, youth SED was the sum of six z-scored variables: father's social class at birth, father's social class at age 7, age at which the father left education, age at which the mother left education, parental housing tenure in childhood, and the number of people sharing a room in the household at age 7. This variable was composed to be consistent with earlier work [47].

Outcome

BMI was assessed 4 times, when the average age was 24, 33, 45, and 53 in the NLSY, and 23, 33, 42, and 55 in the NCDS. BMI was calculated as weight divided by the square of height, and where measurements were taken in Imperial units, they were converted to metric. Values of BMI greater than 70 or less than 12 were treated as biologically implausible [48] and the observations were removed from the dataset. From the NCDS, 0 such cases needed to be removed at age 23, 65 at age 33, 11 at age 42, and 4 at age 55. From the NLSY, only two such cases needed to be removed, at the 1985 wave.

Covariates

Education was measured in age at which an individual left school in the NCDS, and highest grade achieved in the NLSY, which was only converted to age at which an individual left school for descriptive purposes in Table 1. Each variable was independently z-scored for use in our models.

Net family income was used in both samples. Net family income was derived from a comprehensive set of income questions in both samples. The possible sources came from all

For peer review only - http://bmjopen.bmj.com/site/about/guidelines.xhtml

earning members of the household, and included military income, wages, salaries, tips, unemployment compensation, child support, alimony, food stamps, welfare and disability benefits, interest and dividends, and others. The derivation of these variables in the NLSY is documented on the National Longitudinal Surveys website (https://www.nlsinfo.org/content/cohorts/nlsy79/topical-guide/income/income/) and in the NCDS the derivation of these variables was managed by the Centre for Longitudinal Studies[49] (see also Appendix 1). Both income variables were top coded to ensure confidentiality of each sample's top earners, which also protects against some of the effects of large outlying observations.

Latent Growth Curve Modeling

We modeled change in BMI across time using latent growth curve models. Latent growth curve models are a form of structural equation model[50] where, in this case, the growth curve model fits latent variables to observed variables, with the latent variables representing an individual's overall level (intercept) of a variable across time, as well as latent variables for change (slope, or trajectory) over time. These latent variables can be related to other observed variables with conventional regression and covariance methods.

The latent varaibles in our model were derived from 4 BMI measurements taken at roughly decade-long intervals. The BMI latent variables included variables for overall BMI level (or intercept), linear change, and quadratic change. All analyses were carried out using the R package 'lavaan'[51].

Each BMI latent variable was regressed on youth SED, education, sex, cognitive function, and the interaction between sex and cognitive function. BMI measurements were

For peer review only - http://bmjopen.bmj.com/site/about/guidelines.xhtml

regressed on income measurements corresponding to the same measurement time points. Because there was substantially more age variation among NLSY participants, additional control variables were included in those models. These were age at cognitive testing in 1979, upon which was regressed the BMI latent variables, as we did with cognitive function and other time-invariant variables, and age during each follow-up wave, upon which, like income, was regressed the BMI measurements. The variances of the first BMI observations had to be constrained to be greater than 0 in both the NLSY and NCDS models, but this did not substantially impact the fit of either model.

All *P* values are two-side and have been corrected for multiple comparisons using the False Discovery Rate[52] within models. Missing data handling procedures are described in Appendix 2 of the Supplementary Information.

Patient and public involvement

Data were deidentified and no patients were involved in this research. The results of this project will be disseminated via the usual academic media and in the press.

RESULTS

Overview of the results

We first describe the ultimate structure of our latent growth curve models, second we describe the general appearance of the results, and then report the results of the models in each country separately. All models were a variation on the one presented in Figure 1. BMI change over time was best modeled with quadratic and linear slope components in both

For peer review only - http://bmjopen.bmj.com/site/about/guidelines.xhtml

samples (NLSY: χ^2 = 30.743, df = 13, CFI = 0.998, SRMR = 0.009; NCDS: χ^2 = 286.905, df = 1, CFI = 0.985, SRMR = 0.027), suggesting that BMI increases over time, and that the rate of increase generally decreases over time (Table S1). The main results of our models are shown in Table 2, and explained below.

In both the US and the UK, and in men and women, mean BMI increases from the 20s to the 50s, by about 4 to 5 kg/m² (Table 1, Figure 2). Men appear to have higher mean BMI than women (Figure 2, upper panel). Americans have higher BMI than the British from early adulthood, and the differences grow as adulthood advances; Americans were more than half a BMI unit higher than the British when participants were in their 20s, and nearly 1.5 BMI unit higher when participants were in their 40s. Although cognitive function was modelled as a continuum, we show it in Figure 2 as tertiles for descriptive purposes only. In both the US and the UK, throughout the period of adulthood examined, the highest cognitive function tertile has the lowest mean BMI and the lowest tertile has the highest BMI (Figure 2, middle panel). This was seen in both countries, and in men and women (Figure 2, lower panel). Next, we report the results of formal modelling of that data.

United States

In the US sample, men had higher BMI level than women, but there were no main effects of cognitive function, early life sociodemographic characteristics, or education (Table 2). There was evidence for an interaction between sex and cognitive function on BMI; the model's results suggest that cognitive function is not related to BMI level in American men, but, in American women, 1 standard deviation higher cognitive function is associated with a more than half unit lower BMI level.

For peer review only - http://bmjopen.bmj.com/site/about/guidelines.xhtml

We found no associations between early-life cognitive function or sociodemographic variables and rate of BMI increase, although linear BMI change was negatively associated with quadratic BMI change. This suggests that more rapid BMI increases earlier in life are accompanied by a flattening-out of BMI change in middle-age, which is consistent with what we see in our visualizations (Figure 2).

United Kingdom

In the British sample, women had lower BMI levels than men, more than 0.8 units all else being equal (Table 2). Higher cognitive function in youth was associated with lower BMI level; 1 standard deviation higher score in cognitive function was associated with ~0.2 units lower BMI. There was no sex by cognitive function interaction on BMI level. Additionally, and unlike in the American sample, high youth SED and more educational attainment were associated with higher and lower BMI levels, respectively. We note that these coefficients in the US samples are not dissimilar to those in the UK sample, and that the UK sample is larger and probably has greater power.

As found in the American sample, there were no relationships between BMI growth and cognitive function, youth SED, or education. However, there was an association between BMI growth and sex, so that women had slower growth in BMI over time. Every decade, men gained about 0.3 BMI units more than women, all else being equal. Sex was also associated with the quadratic component of BMI growth, such that men's BMI growth tended to level off in later ages, whereas women's linear BMI growth continued more linearly in later ages. These British effects are visible in Figure 2.

In the UK sample, BMI level was associated with linear BMI slope, and linear slope was associated with quadratic slope. This means that higher BMI level indicated greater

For peer review only - http://bmjopen.bmj.com/site/about/guidelines.xhtml

linear growth, and having a higher linear growth component was associated with more flattening out of growth over time. At one point in time, and only in the British sample, income was related to BMI: at age 23, having a higher income was associated with having higher BMI at that age (*B* = 0.574, 95% C.I.: 0.390, 0.758, $P < 0.001$).

Sensitivity Analyses

As our primary interest was in early-life factors, we wished to explore how removing other variables, first income, then education, might influence associations with BMI variables. Moreover, some of these income and education measurements are missing for some participants, so fitting models without them would give us additional power to detect effects, by not requiring that our models rely as heavily on maximum likelihood estimation to fill in the missing values.

Modeling without income (Table S2) allowed us to confirm the association between higher education and lower BMI levels in the US (*B* = -0.339, 95% C.I.: -0.534, -0.143, *P* = 0.004). Associations were unchanged in the UK when income was removed. Going further and removing education (Table S3) revealed associations in the US between SED in youth and BMI level (*B* = 0.274, 95% C.I.: 0.101, 0.448, *P* = 0.010) and BMI slope (*B* = 0.231, 95% C.I.: 0.051, 0.412, *P* = 0.030), such that more disadvantaged Americans have higher overall BMI levels and faster growth. Removing education in the UK model revealed an association between youth SED and quadratic BMI growth (*B* = 0.032, 95% C.I.: 0.004, 0.060, *P* = 0.045), which would suggest that all else being equal, more disadvantaged British participants showed less leveling off of BMI growth over time.

We also wished to see if our findings generalized to the entire populations represented in these samples, so we refit our primary models (i.e. those described in Table 2) with all

For peer review only - http://bmjopen.bmj.com/site/about/guidelines.xhtml

participants from each sample, regardless of their ethnic group. All of the effects previously described persisted with similar effect sizes (Table S4). Cognitive function was associated with higher BMI level across sexes in the United Kingdom, but the protective effect of cognitive function was only present in American women.

DISCUSSION

From their 20s to their 50s, both the US and UK participants had similar starting points and trajectories of growth in BMI. Almost everyone gained weight as they aged. Both populations show steeper BMI growth in their 20s and 30s, and neither sociodemographic nor cognitive factors were associated with these changes. Nevertheless, some factors did predict whether participants would have lower or higher BMI levels that would be reflected from early adulthood into middle age, though these factors often differed between the American and the British samples.

A straightforward source of difference in BMI levels came from sex. Both American and British women have lower BMIs than men. However, American women are likely to have nearly 1.5 fewer BMI units than American men, whereas British women are likely to have only about 0.85 fewer BMI units than British men. A possible reason is visible in Figure 2: Americans weigh more in general and that allows Americans to have greater range of BMI values, so for example, there is more opportunity for American men to have higher BMI than the average American.

In their 20s, the lowest BMI American women approximately matched the lowest BMI British women, but the American sample rapidly gained more weight, and by their 40s, the average American is more than a full BMI unit higher. Diversity in BMI grows across both samples as well: the SD of BMI among Americans goes from 4 units in the early 20s to

For peer review only - http://bmjopen.bmj.com/site/about/guidelines.xhtml

more than 6 in the 50s, and among the British the SD rises from ~3 at age 23 to ~5.5 at age 55. BMI growth is faster in British men than British women, but that growth slowed in participants' 40s and 50s, whereas in British women, growth was slower but more consistent. No such effects were apparent in Americans. Both men and women tended to gain weight at the same rate.

Associations between cognitive function and BMI levels were present in both samples. British with higher cognitive function tended to have lower BMI: a British person of average height (~1.7m) with a 15 IQ point, which is equivalent to 1 SD, lower score than average would likely weigh at least half a kilogram (1.1 lbs.) more than average. On the other hand, only higher cognitive function American women were likely to have lower BMI. For an average American woman (~1.6m), a 15 IQ point (again, 1SD) lower score than average would likely be equated with being 1.3 kilogram heavier (2.9 lbs.). Cognitive function was one of the more noticeable sources of difference in BMI (Figure 2); in the American sample, only cognitive function and sex, not socioeconomic factors, appeared to be associated with BMI.

In addition to cognitive function, education and youth SED were also associated with BMI levels in British people, but not in Americans. Having more education was associated with lower BMI, to nearly the same extent as cognitive function. Coming from a less deprived background was also associated with lower BMI, to a greater degree than either cognitive function or education, though, again, only in the British. In the American sample, education showed a similar effect size and a nearly overlapping confidence interval; therefore, with more participants and thus power, we might have found that education was similarly associated in the American sample. Some, but not all, of our sensitivity analyses support this, so imprecision of variable measurement might also explain the width of this confidence interval. Income was not associated with BMI at any point in our analyses, except

For peer review only - http://bmjopen.bmj.com/site/about/guidelines.xhtml

among the British when they were age 23. At this age, income may be related to BMI in the same way as youth SED, which is composed of parents' sociodemographic variables from a few years earlier.

Particular strengths of this study include that it is longitudinal, with multiple measurements spanning 30 years. Moreover, a valuable and unusual strength is that the variables and their life-course timings in the two countries were remarkably similar. We identified and derived highly analogous variables for every major factor in the study, making the direct comparisons across these two cultures feasible.

There were some limitations to this study as well. For greater comparability between samples, we focused our study on American and British participants of European descent. However, our own sensitivity analyses as well as previous analyses give us no reason to believe that these findings are not applicable in other ethnic groups.[19 38] The NLSY and NCDS samples are now both middle-aged, so our growth curve models may not fit in precisely the same way in other age groups, particularly younger samples, whose early-life background may have been different from what our participants experienced in the 1950s, 60s, and 70s. Additionally, our American sample was smaller than our British sample, so we did not have as much power to detect relationships in Americans.

BMI is an imperfect measure;[53] it does not always effectively capture individual adiposity, nor does it indicate that any particular mechanism is at work. However, we did not have access to repeated measures of other adiposity measures, e.g. waist circumference or % body fat. Nevertheless, BMI is important to study as it remains and will continue to be widely used, and it is a risk factor for many illnesses.[3-6]

Previous work with the NLSY has demonstrated that trajectories of BMI over the same stretches of adulthood are associated with social factors including sex, SED, and

For peer review only - http://bmjopen.bmj.com/site/about/guidelines.xhtml

education.[54 55] Cognitive function in youth has also been linked to maternal and offspring BMI in the NLSY,[20] but to the best of our knowledge, cognitive function has not been associated with trajectories of BMI across the life course. Similarly, studies with British cohorts, including the NCDS, have found that socioeconomic disparities are associated with BMI differences,[56 57] but cognitive function and trajectories of BMI is much less studied. A notable exception is Chandola, et al. [37] who also used LGCMs with the NCDS, but analyzed men and women in different models, thus could not detect the interaction effects we found. Our use of LGCMs allowed us to model change in individuals' BMI over the same period of time, incorporating the same variables, but in distinct cultures. Moreover, our analyses being cross-cultural provides further validation for the effects we found that spanned both of our samples, thus demonstrating that these results are not unique to a particular nation or culture.

What is apparent is from our results is that cognitive function tested in youth has an important association with BMI, from early adulthood into middle-age. BMI and cognitive function are genetically correlated at r_g = -0.13, [58] so some of the association we found might be caused by genetic variation; however, some of the genetic risk of obesity can be reduced through education.[59] It is therefore important to ask: do people learn things over the course of their education that they use to live healthier lives? In much the same way, we might be able to encourage what has been termed "phenocopying"[17] – that is, encouraging and enabling people to follow the same strategies that individuals with higher cognitive function use to look after their health with the hope of achieving the same results. In contrast to education, which we only found to be associated with BMI in the UK, we found associations between cognitive function and BMI in both samples, as did two prior studies.[58 59]

The associations between cognitive function, sociodemographic factors, and BMI were mostly with an individual's overall BMI level. BMI level is thus of particular importance because it indicates whether a person is overweight or obese from the start of

For peer review only - http://bmjopen.bmj.com/site/about/guidelines.xhtml

these samples, and obesity among younger adults is particularly hazardous to one's later life health.[60] Youth SED might be linked to major early life determinants of overweight and obesity,[61] and so, through phenocopying and related health education, early interventions are probably best positioned to make a difference to individuals' lifelong health.

This study shows that BMI growth is similar across two nationalities, in spite of major differences in BMI baselines between the US and UK. Moreover, BMI growth appears to be largely unaffected by cognitive and sociodemographic factors. Baseline BMI levels (rather than their rate of change) are influenced by external factors - cognitive function being one of the strongest; its effect appeared in both the US and UK samples. Identifying the behaviors and other pathways through which cognitive function might protect individuals from overweight and obesity should be investigated further in the pursuit of real-world solutions that can help us reduce the global burden of the overweight and obesity epidemic.

AUTHORSHIP CONTRIBUTIONS

DMA discussed and planned the study and analyses, analysed the data, interpreted the data, and drafted the initial manuscript. CW discussed and planned the study and analyses, interpreted the data and contributed to the manuscript. CRG discussed and planned the study and analyses, interpreted the data and contributed to the manuscript. IJD discussed and planned the study and analyses, interpreted the data and contributed to the manuscript.

For peer review only - http://bmjopen.bmj.com/site/about/guidelines.xhtml

For peer review only

DATA SHARING STATEMENT

Anonymized NCDS data are available from the UK Data Service at https://www.ukdataservice.ac.uk/. Anonymized NLSY data are available online from the US Bureau of Labor Statistics at https://www.nlsinfo.org/investigator/.

FUNDING STATEMENT

This work was supported by the University of Edinburgh Centre for Cognitive Ageing and Cognitive Epidemiology, part of the cross council Lifelong Health and Wellbeing Initiative (MR/K026992/1). Funding from the Biotechnology and Biological Sciences Research Council (BBSRC), Economic and Social Research Council (ESRC) and Medical Research Council (MRC) is gratefully acknowledged. This work was also supported by an MRC Mental Health Data Pathfinder award (MC_PC_17209).

COMPETING INTERESTS

All authors declare that they have no competing interests.

For peer review only

References

1. Roberto CA, Swinburn B, Hawkes C, et al. Patchy progress on obesity prevention: emerging examples, entrenched barriers, and new thinking. *The Lancet* 2015;385(9985):2400-09.
2. Collaborators GO. Health effects of overweight and obesity in 195 countries over 25 years. *New Engl J Med* 2017;377(1):13-27.
3. Bogers RP, Bemelmans WJ, Hoogenveen RT, et al. Association of overweight with increased risk of coronary heart disease partly independent of blood pressure and cholesterol levels: a meta-analysis of 21 cohort studies including more than 300 000 persons. *Archives of internal medicine* 2007;167(16):1720-28.
4. Renehan AG, Tyson M, Egger M, et al. Body-mass index and incidence of cancer: a systematic review and meta-analysis of prospective observational studies. *The Lancet* 2008;371(9612):569-78.
5. Guh DP, Zhang W, Bansback N, et al. The incidence of co-morbidities related to obesity and overweight: a systematic review and meta-analysis. *BMC Public Health* 2009;9(1):88.
6. Luppino FS, de Wit LM, Bouvy PF, et al. Overweight, obesity, and depression: a systematic review and meta-analysis of longitudinal studies. *Archives of general psychiatry* 2010;67(3):220-29.
7. De Wit L, Luppino F, van Straten A, et al. Depression and obesity: a meta-analysis of community-based studies. *Psychiatry research* 2010;178(2):230-35.
8. Gariepy G, Nitka D, Schmitz N. The association between obesity and anxiety disorders in the population: a systematic review and meta-analysis. *Int J Obesity* 2010;34(3):407.
9. Zhao Z, Okusaga OO, Quevedo J, et al. The potential association between obesity and bipolar disorder: a meta-analysis. *Journal of affective disorders* 2016;202:120-23.
10. Heid IM, Jackson AU, Randall JC, et al. Meta-analysis identifies 13 new loci associated with waist-hip ratio and reveals sexual dimorphism in the genetic basis of fat distribution. *Nat Genet* 2010;42(11):949.
11. Farooqi I, O'Rahilly S. New advances in the genetics of early onset obesity: Nature Publishing Group, 2005.
12. Shrewsbury V, Wardle J. Socioeconomic status and adiposity in childhood: a systematic review of cross-sectional studies 1990–2005. *Obesity* 2008;16(2):275-84.
13. Danese A, Tan M. Childhood maltreatment and obesity: systematic review and meta-analysis. *Mol Psychiatry* 2014;19(5):544.
14. Taveras EM, Gillman MW, Kleinman K, et al. Racial/Ethnic Differences in Early-Life Risk Factors for Childhood Obesity. *Pediatrics* 2010;125(4):686.
15. Wang Y, Beydoun MA. The obesity epidemic in the United States—gender, age, socioeconomic, racial/ethnic, and geographic characteristics: a systematic review and meta-regression analysis. *Epidemiol Rev* 2007;29(1):6-28.
16. Koenen KC, Moffitt TE, Roberts AL, et al. Childhood IQ and adult mental disorders: a test of the cognitive reserve hypothesis. *Am J Psychiatry* 2009;166(1):50-57.
17. Deary IJ, Weiss A, Batty GD. Intelligence and personality as predictors of illness and death: How researchers in differential psychology and chronic disease epidemiology are collaborating to understand and address health inequalities. *Psychol Sci Publ Int* 2010;11:53-79. doi: 10.1177/1529100610387081
18. Calvin CM, Batty GD, Der G, et al. Childhood intelligence in relation to major causes of death in 68 year follow-up: prospective population study. *bmj* 2017;357:j2708.
19. Wraw C, Deary IJ, Gale CR, et al. Intelligence in youth and health at age 50. *Intelligence* 2015;53:23-32.
20. Wraw C, Deary IJ, Der G, et al. Maternal and offspring intelligence in relation to BMI across childhood and adolescence. *Int J Obesity* 2018 doi: 10.1038/s41366-018-0009-1
21. Wraw C, Deary IJ, Der G, et al. Intelligence in youth and mental health at age 50. *Intelligence* 2016;58:69-79.

For peer review only - http://bmjopen.bmj.com/site/about/guidelines.xhtml

22. Kanazawa S. Intelligence and obesity: which way does the causal direction go? *Current Opinion in Endocrinology, Diabetes and Obesity* 2014;21(5):339-44.
23. Batty G, Gale C, Mortensen LH, et al. Pre-morbid intelligence, the metabolic syndrome and mortality: the Vietnam Experience Study. *Diabetologia* 2008;51(3):436-43.
24. Belsky DW, Caspi A, Goldman-Mellor S, et al. Is obesity associated with a decline in intelligence quotient during the first half of the life course? *Am J Epidemiol* 2013;178(9):1461-68.
25. Verhaeghen P, Salthouse TA. Meta-analyses of age–cognition relations in adulthood: Estimates of linear and nonlinear age effects and structural models. *Psychological bulletin* 1997;122(3):231.
26. Cheng G, Huang C, Deng H, et al. Diabetes as a risk factor for dementia and mild cognitive impairment: a meta-analysis of longitudinal studies. *Intern Med J* 2012;42(5):484-91. doi: 10.1111/j.1445-5994.2012.02758.x
27. Lang CC, Mancini DM. Non-cardiac comorbidities in chronic heart failure. *Heart* 2007;93(6):665-71.
28. Snyder HR. Major depressive disorder is associated with broad impairments on neuropsychological measures of executive function: a meta-analysis and review. *Psychological bulletin* 2013;139(1):81.
29. Profenno LA, Porsteinsson AP, Faraone SV. Meta-analysis of Alzheimer's disease risk with obesity, diabetes, and related disorders. *Biological psychiatry* 2010;67(6):505-12.
30. Chen J, Guan Z, Wang L, et al. Meta-analysis: overweight, obesity, and Parkinson's disease. *International journal of endocrinology* 2014;2014
31. Beydoun MA, Beydoun H, Wang Y. Obesity and central obesity as risk factors for incident dementia and its subtypes: a systematic review and meta-analysis. *Obesity reviews* 2008;9(3):204-18.
32. Wardle J, Waller J, Jarvis MJ. Sex differences in the association of socioeconomic status with obesity. *Am J Public Health* 2002;92(8):1299-304.
33. Kavanagh A, Bentley RJ, Turrell G, et al. Socioeconomic position, gender, health behaviours and biomarkers of cardiovascular disease and diabetes. *Social science & medicine* 2010;71(6):1150-60.
34. Thurston RC, Kubzansky LD, Kawachi I, et al. Is the association between socioeconomic position and coronary heart disease stronger in women than in men? *Am J Epidemiol* 2005;162(1):57-65.
35. Lawlor DA, Batty GD, Clark H, et al. Association of childhood intelligence with risk of coronary heart disease and stroke: findings from the Aberdeen Children of the 1950s cohort study. *Eur J Epidemiol* 2008;23(10):695-706.
36. Leng B, Jin Y, Li G, et al. Socioeconomic status and hypertension: a meta-analysis. *Journal of hypertension* 2015;33(2):221-29.
37. Chandola T, Deary IJ, Blane D, et al. Childhood IQ in relation to obesity and weight gain in adult life: the National Child Development (1958) Study. *Int J Obesity* 2006;30(9):1422.
38. Altschul DM, Wraw C, Der G, et al. Hypertension development by midlife and the roles of pre-morbid cognitive function, sex, and their interaction. *Hypertension* 2019
39. Bell CG, Walley AJ, Froguel P. The genetics of human obesity. *Nature reviews genetics* 2005;6(3):221.
40. Carmienke S, Freitag M, Pischon T, et al. General and abdominal obesity parameters and their combination in relation to mortality: a systematic review and meta-regression analysis. *European journal of clinical nutrition* 2013;67(6):573.
41. Palmer P, Hartke DD, Ree MJ, et al. Armed Services Vocational Aptitude Battery (ASVAB): Alternate Forms Reliability (Forms 8, 9, 10, and 11): AIR FORCE HUMAN RESOURCES LAB BROOKS AFB TX, 1988.
42. Welsh Jr JR, Kucinkas SK, Curran LT. Armed services vocational battery (ASVAB): Integrative review of validity studies: OPERATIONAL TECHNOLOGIES CORP SAN ANTONIO TX, 1990.

For peer review only

For peer review only - http://bmjopen.bmj.com/site/about/guidelines.xhtml

552 43. Der G, Batty GD, Deary IJ. The association between IQ in adolescence and a range of health outcomes at 40 in the 1979 US National Longitudinal Study of Youth. *Intelligence* 2009;37(6):573-80.
555 44. Herrnstein RJ, Murray C. Bell curve: Intelligence and class structure in American life: Simon and Schuster 2010.
557 45. Pigeon D. Tests used in the 1954 and 1957 surveys. *The home and the school* 1964:129-32.
558 46. Douglas JWB. The home and the school: MacGibbon & Kee London 1964.
559 47. Bridger E, Daly M. Does cognitive ability buffer the link between childhood disadvantage and adult health? *Health Psychol* 2017;36(10):966.
561 48. Li W, Kelsey JL, Zhang Z, et al. Small-area estimation and prioritizing communities for obesity control in Massachusetts. *Am J Public Health* 2009;99(3):511-19.
563 49. Goodman A, Joyce R, Smith JP. The long shadow cast by childhood physical and mental problems on adult life. *Proceedings of the National Academy of Sciences* 2011:201016970.
565 50. Preacher KJ. Latent growth curve modeling: Sage 2008.
566 51. Rosseel Y. lavaan: an R package for structural equation modeling and more Version 0.4-9 (BETA): Ghent University, 2011.
568 52. Benjamini Y, Hochberg Y. Controlling the false discovery rate: A practical and powerful approach to multiple testing. *Journal of the Royal Statistical Society: Series B (Methodological)* 1995;57:289-300.
571 53. Gómez-Ambrosi J, Silva C, Galofré J, et al. Body mass index classification misses subjects with increased cardiometabolic risk factors related to elevated adiposity. *Int J Obesity* 2012;36(2):286.
574 54. Østbye T, Malhotra R, Landerman LR. Body mass trajectories through adulthood: results from the National Longitudinal Survey of Youth 1979 Cohort (1981–2006). *Int J Epidemiol* 2010;40(1):240-50. doi: 10.1093/ije/dyq142
577 55. Benson R, von Hippel PT, Lynch JL. Does more education cause lower BMI, or do lower-BMI individuals become more educated? Evidence from the National Longitudinal Survey of Youth 1979. *Social Science & Medicine* 2018;211:370-77.
580 56. Bann D, Johnson W, Li L, et al. Socioeconomic Inequalities in Body Mass Index across Adulthood: Coordinated Analyses of Individual Participant Data from Three British Birth Cohort Studies Initiated in 1946, 1958 and 1970. *PLOS Medicine* 2017;14(1):e1002214. doi: 10.1371/journal.pmed.1002214
584 57. Bann D, Johnson W, Li L, et al. Socioeconomic inequalities in childhood and adolescent body-mass index, weight, and height from 1953 to 2015: an analysis of four longitudinal, observational, British birth cohort studies. *The Lancet Public Health* 2018;3(4):e194-e203. doi: https://doi.org/10.1016/S2468-2667(18)30045-8
588 58. Davies G, Lam M, Harris SE, et al. Study of 300,486 individuals identifies 148 independent genetic loci influencing general cognitive function. *Nature communications* 2018;9(1):2098.
590 59. Barcellos SH, Carvalho LS, Turley P. Education can reduce health differences related to genetic risk of obesity. *Proceedings of the National Academy of Sciences* 2018;115(42):E9765.
592 60. Fontaine KR, Redden DT, Wang C, et al. Years of life lost due to obesity. *JAMA* 2003;289(2):187-93. doi: 10.1001/jama.289.2.187
594 61. Monasta L, Batty G, Cattaneo A, et al. Early-life determinants of overweight and obesity: a review of systematic reviews. *Obesity reviews* 2010;11(10):695-708.

For peer review only - http://bmjopen.bmj.com/site/about/guidelines.xhtml

597 **Figure 1. Path diagram of latent growth curve models used in primary analyses.** Square
598 boxes are observed variables, circles are latent variables. Single headed arrows are
599 regressions or latent variable loadings, double headed arrows are covariances.

600

601 **Figure 2. Quadratic growth curves of BMI fitted to tertiles of cognitive function, and**
602 **across different sexes.** The top panels illustrates BMI between sexes, the middle panels
603 illustrates BMI in different general cognitive function (g) tertiles, and the bottom panels
604 illustrate the interaction between cognitive function tertiles and sex. Shaded areas represent
605 the 95% error regions.

606

607

608

609

610

611

612

613

614

615

616

617

For peer review only

For peer review only - http://bmjopen.bmj.com/site/about/guidelines.xhtml

Table 1. Descriptive statistics for all tested variables.

	NLSY - United States				NCDS - United Kingdom			
Variable		N	Median	SD		N	Median	SD
BMI (kg/m²)	average age				average age			
	24	6198	22.71	4.00	23	9835	22.13	3.09
	33	4254	24.96	4.98	33	8741	24.62	4.57
	45	3651	26.62	5.53	42	8787	25.43	4.66
	53	3396	27.77	6.04	55	6861	26.79	5.49
Net family income ($ or £)								
	24	5386	$382	$365.17	23	10027	£87	£52.25
	33	3709	$769	$766.83	33	9094	£272	£178.64
	45	3593	$1288	$1624.87	42	9032	£435	£399.02
	53	3092	$1415	$2133.50	50	7846	£600	£554.46
Cognitive function		7023	0.12			11571	0.13	
Youth SED		7023	0.01			12290	0.15	
Education		3788	-0.03			8819	-0.53	
Sex			3787 male	3719 female			5633 male	5320 female

NLSY = National Longitudinal Study of Youth 1979, NCDS = National Child Development Study, SED = Socioeconomic Disadvantage. Cognitive function, youth SED, and education, are unitless variables, thus they are scaled so that their SD is 1. The means are 0, so the medians indicate the amount of skewness in the distributions.

For peer review only

For peer review only - http://bmjopen.bmj.com/site/about/guidelines.xhtml

Table 2. Coefficient estimates from models of BMI growth, cognitive function, and sociodemographic variables.

		United States				United Kingdom			
Outcome	Predictor	Estimate	95%	C.I.	*P*	Estimate	95%	C.I.	*P*
BMI Level	Sex (women)	**-1.444**	**[-1.820,**	**-1.068]**	**< 0.001**	**-0.849**	**[-1.016,**	**-0.681]**	**< 0.001**
	Cognitive function	0.105	[-0.221,	0.431]	0.683	**-0.196**	**[-0.330,**	**-0.063]**	**0.011**
	Sex x Cognitive function	**-0.525**	**[-0.901,**	**-0.149]**	**0.046**	-0.053	[-0.222,	0.116]	0.694
	Youth SED	0.178	[-0.047,	0.403]	0.348	**0.332**	**[0.230,**	**0.434]**	**< 0.001**
	Education	-0.248	[-0.497,	0.001]	0.225	**-0.153**	**[-0.263,**	**-0.042]**	**0.017**
BMI Slope	Sex (women)	0.157	[-0.230,	0.544]	0.670	**-0.327**	**[-0.504,**	**-0.150]**	**0.001**
	Cognitive function	0.092	[-0.254,	0.438]	0.733	0.036	[-0.110,	0.182]	0.694
	Sex x Cognitive function	0.295	[-0.087,	0.677]	0.348	-0.140	[-0.322,	0.041]	0.239
	Youth SED	0.281	[0.045,	0.518]	0.108	0.028	[-0.084,	0.140]	0.694
	Education	-0.082	[-0.332,	0.168]	0.683	0.030	[-0.082,	0.143]	0.694
	BMI Level	-0.319	[-0.810,	0.171]	0.443	**0.384**	**[0.184,**	**0.584]**	**0.001**
BMI Quadratic	Sex (women)	0.043	[-0.085,	0.172]	0.683	**0.135**	**[0.080,**	**0.189]**	**< 0.001**
	Cognitive function	-0.003	[-0.119,	0.113]	0.978	-0.009	[-0.054,	0.037]	0.742
	Sex x Cognitive function	-0.095	[-0.222,	0.032]	0.348	0.026	[-0.031,	0.084]	0.541
	Youth SED	-0.038	[-0.118,	0.043]	0.627	0.027	[-0.009,	0.062]	0.242
	Education	0.020	[-0.063,	0.104]	0.733	-0.015	[-0.051,	0.021]	0.563
	BMI Level	-0.002	[-0.166,	0.161]	0.978	-0.071	[-0.139,	-0.003]	0.083
	BMI Slope	-1.554	**[-1.810,**	**-1.298]**	**< 0.001**	**-1.006**	**[-1.146,**	**-0.866]**	**< 0.001**
χ2		83.966				137.465			
df		31				18			
CFI		0.992				0.991			
SRMR		0.015				0.011			

Models are latent growth curve models, with latent variables for BMI level, slope, and quadratic slope - see the path diagram in Figure 1. The same path diagram was used for both samples, except the US sample was modeled with adjustments for individual ages. All coeffecient values are multiple regression coefficients from the predictor onto the outcome, except the associations between the three BMI latent variables, which are

For peer review only

For peer review only - http://bmjopen.bmj.com/site/about/guidelines.xhtml

covariances. Effect size estimates are for 1SD changes in a variable. P-values are corrected for multiple comparisons using the false discovery rate. SED = socioeconomic disadvantage.

621

For peer review only

For peer review only - http://bmjopen.bmj.com/site/about/guidelines.xhtml

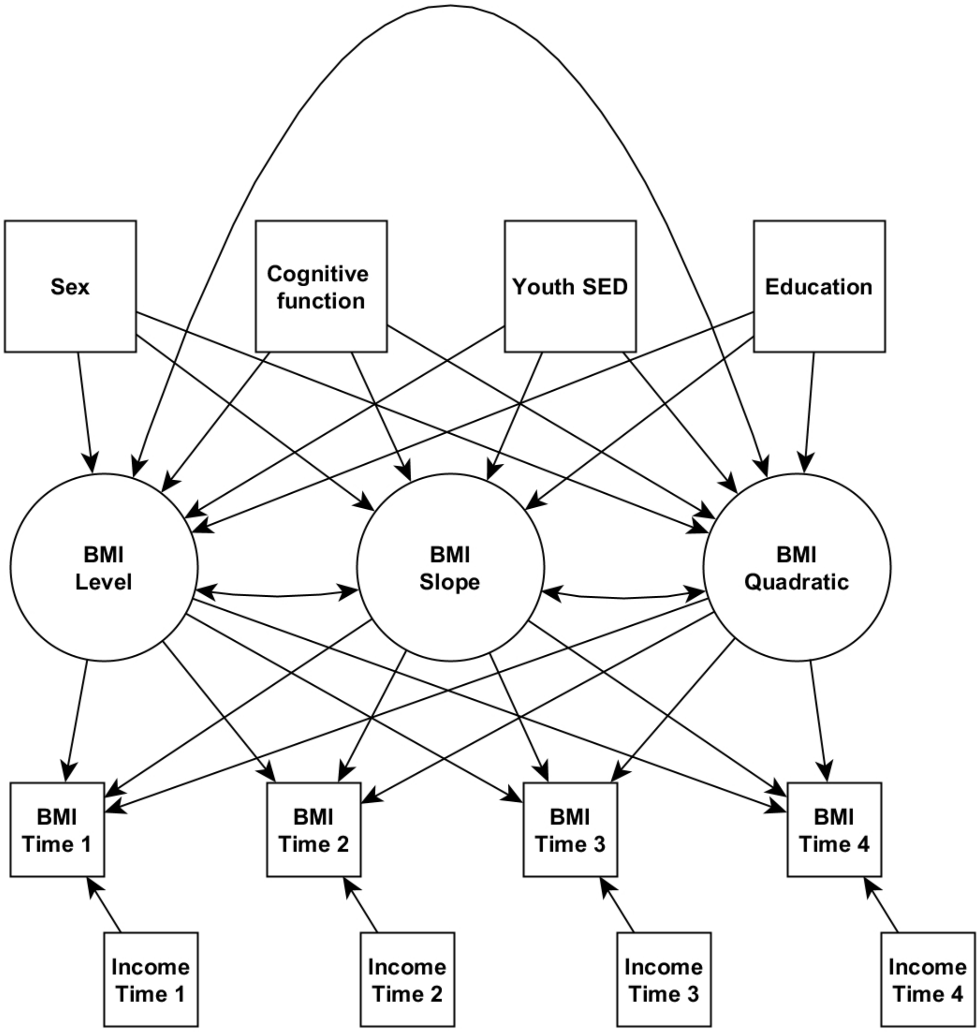

Figure 1. Path diagram of latent growth curve models used in primary analyses. Square boxes are observed variables, circles are latent variables. Single headed arrows are regressions or latent variable loadings, double headed arrows are covariances.

376x395mm (72 x 72 DPI)

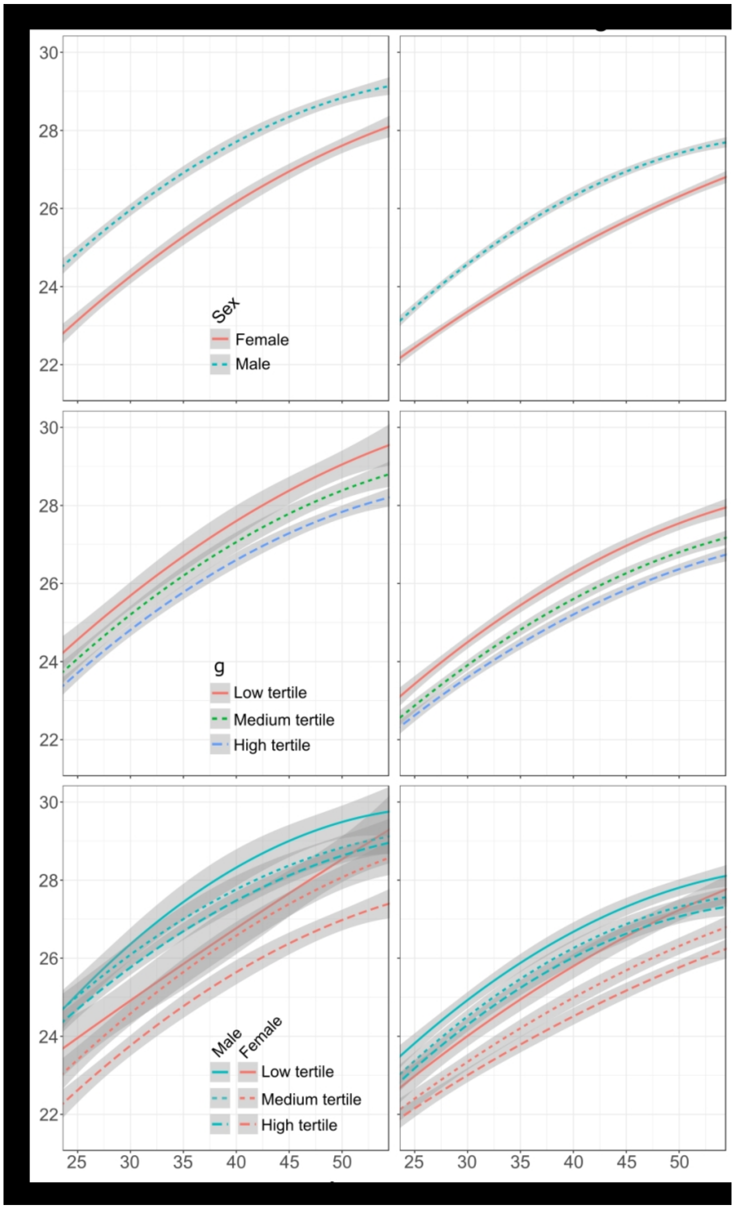

Figure 2. Quadratic growth curves of BMI, United States on the left, United Kingdom on the right, fitted to tertiles of cognitive function, and across different sexes. The top panels illustrates BMI between sexes, the middle panels illustrates BMI in different general cognitive function (g) tertiles, and the bottom panels illustrate the interaction between cognitive function tertiles and sex. Shaded areas represent the 95% error regions.

Supplementary Information for

How early-life cognitive and sociodemographic factors relate to the development of overweight and obesity in the UK and the US: a prospective cross-cohort study of the National Child Development Study and National Longitudinal Study of Youth 1979

For peer review only

This file includes:

Appendix 1 – Methods – Income and measurement time
Appendix 2 – Methods – Missing Data
Supplementary Tables S1 to S4

Appendix 1

Methods – Income and measurement time

Income variables were taken from the same wave as BMI variables with one exception. In the NCDS, income data were collected using a different instrument at age 55, the most recent available time point for BMI data. The age 55 measurement was missing more data than the age 50 measurement (652 more cases), and in addition to there being data collection inconsistencies at age 55, the variable had some problematic properties, e.g. smaller mean and median income statistics compared to those at age 50. Considering that model fit was sub-par using the age 55 measure, but much better using the age 50 measure, we used the age 50 income measure in all of the analyses we present.

For peer review only

Appendix 2

Methods - Missing Data

Study attrition and occasional non-response were present in both samples (see Table 1), though one of the benefits of our approach was that we could grapple with missing data with Full Information Maximum Likelihood estimation using observed information [11]. Standard errors were calculated with first order derivatives and *P*-values were calculated with a χ^2 test, both of which are procedures that can handle missing data.

Prior studies have used multiple imputation to test the robustness of analyses using some of these data [12 13]. They found the same results when comparing imputed and non-imputed model results.

For peer review only

For peer review only - http://bmjopen.bmj.com/site/about/guidelines.xhtml

Table S1. Full table of parameter estimates from each latent growth curve model of BMI growth in the US and UK.

United States				United Kingdom			
LHS	RHS	Estimate	SE	LHS	RHS	Estimate	SE
Latent Variables							
BMI Level	BMI ('85)	1		BMI Level	BMI (age 23)	1	
BMI Level	BMI ('94)	1		BMI Level	BMI (age 33)	1	
BMI Level	BMI ('06)	1		BMI Level	BMI (age 42)	1	
BMI Level	BMI ('14)	1		BMI Level	BMI (age 55)	1	
BMI Slope	BMI ('85)	0		BMI Slope	BMI (age 23)	0	
BMI Slope	BMI ('94)	0.9		BMI Slope	BMI (age 33)	1	
BMI Slope	BMI ('06)	2.1		BMI Slope	BMI (age 42)	1.9	
BMI Slope	BMI ('14)	2.9		BMI Slope	BMI (age 55)	3.2	
BMI Quadratic	BMI ('85)	0		BMI Quadratic	BMI (age 23)	0	
BMI Quadratic	BMI ('94)	0.81		BMI Quadratic	BMI (age 33)	1	
BMI Quadratic	BMI ('06)	4.41		BMI Quadratic	BMI (age 42)	3.61	
BMI Quadratic	BMI ('14)	8.41		BMI Quadratic	BMI (age 55)	10.24	
Regressions							
BMI Level	Sex (women)	-1.444	0.192	BMI Level	Sex (women)	-0.849	0.085
BMI Level	Cognitive function	0.105	0.166	BMI Level	Cognitive function	-0.196	0.068
BMI Level	Sex x Cognitive function	-0.525	0.192	BMI Level	Sex x Cognitive function	-0.053	0.086
BMI Level	Age ('79)	-0.320	1.399				
BMI Level	Youth SED	0.178	0.115	BMI Level	Youth SED	0.332	0.052
BMI Level	Education	-0.248	0.127	BMI Level	Education	-0.153	0.056
BMI Slope	Sex (women)	0.157	0.198	BMI Slope	Sex (women)	-0.327	0.090
BMI Slope	Cognitive function	0.092	0.177	BMI Slope	Cognitive function	0.036	0.075
BMI Slope	Sex x Cognitive function	0.295	0.195	BMI Slope	Sex x Cognitive function	-0.140	0.093
BMI Slope	Age ('79)	1.754	1.725				

For peer review only

For peer review only - http://bmjopen.bmj.com/site/about/guidelines.xhtml

BMI Slope	Youth SED	0.281	0.121	BMI Slope	Youth SED	0.028	0.057
BMI Slope	Education	-0.082	0.128	BMI Slope	Education	0.030	0.057
BMI Quadratic	Sex (women)	0.043	0.066	BMI Quadratic	Sex (women)	0.135	0.028
BMI Quadratic	Cognitive function	-0.003	0.059	BMI Quadratic	Cognitive function	-0.009	0.023
BMI Quadratic	Sex x Cognitive function	-0.095	0.065	BMI Quadratic	Sex x Cognitive function	0.026	0.029
BMI Quadratic	Age ('79)	-0.610	0.570				
BMI Quadratic	Youth SED	-0.038	0.041	BMI Quadratic	Youth SED	0.027	0.018
BMI Quadratic	Education	0.020	0.043	BMI Quadratic	Education	-0.015	0.018
BMI ('85)	Age ('85)	1.717	1.358				
BMI ('85)	Income ('85)	-0.098	0.110	BMI (age 23)	Income (age 23)	0.574	0.094
BMI ('94)	Age ('94)	-0.146	1.107				
BMI ('94)	Income ('94)	0.004	0.101	BMI (age 33)	Income (age 33)	-0.185	0.088
BMI ('06)	Age ('06)	-0.664	1.100				
BMI ('06)	Income ('06)	-0.076	0.076	BMI (age 42)	Income (age 42)	0.015	0.047
BMI ('14)	Age ('14)	-0.324	1.584				
BMI ('14)	Income ('14)	-0.142	0.080	BMI (age 55)	Income (age 50)	0.071	0.058
Variances and covariances							
BMI ('85)	BMI ('85)	0.010	0.000	BMI (age 23)	BMI (age 23)	0.010	#N/A
BMI ('94)	BMI ('94)	4.759	0.156	BMI (age 33)	BMI (age 33)	7.578	0.063
BMI ('06)	BMI ('06)	3.603	0.183	BMI (age 42)	BMI (age 42)	2.364	0.087
BMI ('14)	BMI ('14)	5.745	0.514	BMI (age 55)	BMI (age 55)	5.762	0.677
BMI Level	BMI Level	15.309	0.371	BMI Level	BMI Level	9.064	0.124
BMI Slope	BMI Slope	7.299	0.359	BMI Slope	BMI Slope	5.183	0.186
BMI Quadratic	BMI Quadratic	0.436	0.057	BMI Quadratic	BMI Quadratic	0.255	0.034
BMI Level	BMI Slope	-0.319	0.250	BMI Level	BMI Slope	0.384	0.102
BMI Level	BMI Quadratic	-0.002	0.083	BMI Level	BMI Quadratic	-0.071	0.035
BMI Slope	BMI Quadratic	-1.554	0.131	BMI Slope	BMI Quadratic	-1.006	0.071
Sex (women)	Sex (women)	0.250	0.000	Sex (women)	Sex (women)	0.249	0.000

For peer review only - http://bmjopen.bmj.com/site/about/guidelines.xhtml

For peer review only

Sex (women)	Cognitive function	-0.041	0.000	Sex (women)	Cognitive function	0.031	0.000
Sex (women)	Sex x Cognitive function	0.052	0.000	Sex (women)	Sex x Cognitive function	0.064	0.000
Sex (women)	Age ('79)	0.003	0.000				
Sex (women)	Youth SED	0.020	0.000	Sex (women)	Youth SED	0.011	0.000
Sex (women)	Education	0.002	0.000	Sex (women)	Education	-0.011	0.000
Sex (women)	Age ('85)	0.004	0.000				
Sex (women)	Income ('85)	-0.017	0.000	Sex (women)	Income (age 23)	0.013	0.000
Sex (women)	Age ('94)	0.003	0.000				
Sex (women)	Income ('94)	-0.015	0.000	Sex (women)	Income (age 33)	0.004	0.000
Sex (women)	Age ('06)	0.004	0.000				
Sex (women)	Income ('06)	-0.045	0.000	Sex (women)	Income (age 42)	-0.025	0.000
Sex (women)	Age ('14)	0.003	0.000				
Sex (women)	Income ('14)	-0.094	0.000	Sex (women)	Income (age 50)	-0.053	0.000
Cognitive function	Cognitive function	0.800	0.000	Cognitive function	Cognitive function	0.862	0.000
Cognitive function	Sex x Cognitive function	0.344	0.000	Cognitive function	Sex x Cognitive function	0.442	0.000
Cognitive function	Age ('79)	0.031	0.000				
Cognitive function	Youth SED	-0.375	0.000	Cognitive function	Youth SED	-0.338	0.000
Cognitive function	Education	0.536	0.000	Cognitive function	Education	0.369	0.000
Cognitive function	Age ('85)	0.031	0.000				
Cognitive function	Income ('85)	0.073	0.000	Cognitive function	Income (age 23)	0.013	0.000
Cognitive function	Age ('94)	0.030	0.000				
Cognitive function	Income ('94)	0.228	0.000	Cognitive function	Income (age 33)	0.103	0.000
Cognitive function	Age ('06)	0.028	0.000				
Cognitive function	Income ('06)	0.353	0.000	Cognitive function	Income (age 42)	0.147	0.000
Cognitive function	Age ('14)	0.030	0.000				
Cognitive function	Income ('14)	0.483	0.000	Cognitive function	Income (age 50)	0.206	0.000
Sex x Cognitive function	Sex x Cognitive function	0.363	0.000	Sex x Cognitive function	Sex x Cognitive function	0.451	0.000
Sex x Cognitive function	Age ('79)	0.014	0.000				

For peer review only - http://bmjopen.bmj.com/site/about/guidelines.xhtml

Sex x Cognitive function	Youth SED	-0.163	0.000	Sex x Cognitive function	Youth SED	-0.185	0.000
Sex x Cognitive function	Education	0.236	0.000	Sex x Cognitive function	Education	0.176	0.000
Sex x Cognitive function	Age ('85)	0.013	0.000				
Sex x Cognitive function	Income ('85)	0.034	0.000	Sex x Cognitive function	Income (age 23)	0.017	0.000
Sex x Cognitive function	Age ('94)	0.014	0.000				
Sex x Cognitive function	Income ('94)	0.100	0.000	Sex x Cognitive function	Income (age 33)	0.050	0.000
Sex x Cognitive function	Age ('06)	0.012	0.000				
Sex x Cognitive function	Income ('06)	0.159	0.000	Sex x Cognitive function	Income (age 42)	0.070	0.000
Sex x Cognitive function	Age ('14)	0.013	0.000				
Sex x Cognitive function	Income ('14)	0.185	0.000	Sex x Cognitive function	Income (age 50)	0.089	0.000
Age ('79)	Age ('79)	0.049	0.000				
Age ('79)	Youth SED	-0.005	0.000				
Age ('79)	Education	-0.001	0.000				
Age ('79)	Age ('85)	0.049	0.000				
Age ('79)	Income ('85)	0.011	0.000				
Age ('79)	Age ('94)	0.049	0.000				
Age ('79)	Income ('94)	0.012	0.000				
Age ('79)	Age ('06)	0.049	0.000				
Age ('79)	Income ('06)	0.001	0.000				
Age ('79)	Age ('14)	0.048	0.000				
Age ('79)	Income ('14)	-0.004	0.000				
Youth SED	Youth SED	0.760	0.000	Youth SED	Youth SED	0.909	0.000
Youth SED	Education	-0.418	0.000	Youth SED	Education	-0.397	0.000
Youth SED	Age ('85)	-0.006	0.000				
Youth SED	Income ('85)	-0.082	0.000	Youth SED	Income (age 23)	0.012	0.000
Youth SED	Age ('94)	-0.006	0.000				
Youth SED	Income ('94)	-0.176	0.000	Youth SED	Income (age 33)	-0.080	0.000
Youth SED	Age ('06)	-0.004	0.000				

For peer review only

Youth SED	Income ('06)	-0.278	0.000	Youth SED	Income (age 42)	-0.151	0.000
Youth SED	Age ('14)	-0.006	0.000				
Youth SED	Income ('14)	-0.378	0.000	Youth SED	Income (age 50)	-0.194	0.000
Education	Education	0.937	0.000	Education	Education	0.982	0.000
Education	Age ('85)	0.000	0.000				
Education	Income ('85)	0.066	0.000	Education	Income (age 23)	-0.033	0.000
Education	Age ('94)	-0.001	0.000				
Education	Income ('94)	0.242	0.000	Education	Income (age 33)	0.100	0.000
Education	Age ('06)	-0.001	0.000				
Education	Income ('06)	0.400	0.000	Education	Income (age 42)	0.180	0.000
Education	Age ('14)	0.000	0.000				
Education	Income ('14)	0.552	0.000	Education	Income (age 50)	0.249	0.000
Age ('85)	Age ('85)	0.050	0.000				
Age ('85)	Income ('85)	0.011	0.000				
Age ('85)	Age ('94)	0.048	0.000				
Age ('85)	Income ('94)	0.013	0.000				
Age ('85)	Age ('06)	0.049	0.000				
Age ('85)	Income ('06)	0.002	0.000				
Age ('85)	Age ('14)	0.048	0.000				
Age ('85)	Income ('14)	-0.003	0.000				
Income ('85)	Income ('85)	0.269	0.000	Income (age 23)	Income (age 23)	0.082	0.000
Income ('85)	Age ('94)	0.011	0.000				
Income ('85)	Income ('94)	0.101	0.000	Income (age 23)	Income (age 33)	0.024	0.000
Income ('85)	Age ('06)	0.011	0.000				
Income ('85)	Income ('06)	0.111	0.000	Income (age 23)	Income (age 42)	0.038	0.000
Income ('85)	Age ('14)	0.011	0.000				
Income ('85)	Income ('14)	0.140	0.000	Income (age 23)	Income (age 50)	0.034	0.000
Age ('94)	Age ('94)	0.050	0.000				

For peer review only - http://bmjopen.bmj.com/site/about/guidelines.xhtml

Age ('94)	Income ('94)	0.012	0.000				
Age ('94)	Age ('06)	0.048	0.000				
Age ('94)	Income ('06)	0.002	0.000				
Age ('94)	Age ('14)	0.048	0.000				
Age ('94)	Income ('14)	-0.004	0.000				
Income ('94)	Income ('94)	0.450	0.000	Income (age 33)	Income (age 33)	0.287	0.000
Income ('94)	Age ('06)	0.012	0.000				
Income ('94)	Income ('06)	0.388	0.000	Income (age 33)	Income (age 42)	0.155	0.000
Income ('94)	Age ('14)	0.012	0.000				
Income ('94)	Income ('14)	0.446	0.000	Income (age 33)	Income (age 50)	0.170	0.000
Age ('06)	Age ('06)	0.049	0.000				
Age ('06)	Income ('06)	0.001	0.000				
Age ('06)	Age ('14)	0.048	0.000				
Age ('06)	Income ('14)	-0.005	0.000				
Income ('06)	Income ('06)	1.045	0.000	Income (age 42)	Income (age 42)	0.628	0.000
Income ('06)	Age ('14)	0.001	0.000				
Income ('06)	Income ('14)	0.930	0.000	Income (age 42)	Income (age 50)	0.343	0.000
Age ('14)	Age ('14)	0.049	0.000				
Age ('14)	Income ('14)	-0.002	0.000				
Income ('14)	Income ('14)	1.643	0.000	Income (age 50)	Income (age 50)	0.937	0.000
Intercepts							
BMI ('85)		0.000	0.000	BMI (age 23)		0.000	0.000
BMI ('94)		0.000	0.000	BMI (age 33)		0.000	0.000
BMI ('06)		0.000	0.000	BMI (age 42)		0.000	0.000
BMI ('14)		0.000	0.000	BMI (age 55)		0.000	0.000
Sex (women)		0.511	0.000	Sex (women)		0.530	0.000
Cognitive function		0.287	0.000	Cognitive function		0.199	0.000
Sex x Cognitive function		0.106	0.000	Sex x Cognitive function		0.136	0.000

For peer review only - http://bmjopen.bmj.com/site/about/guidelines.xhtml

For peer review only

Age ('79)	1.807	0.000			
Youth SED	-0.206	0.000	Youth SED	-0.087	0.000
Education	0.303	0.000	Education	0.041	0.000
Age ('85)	2.353	0.000			
Income ('85)	-0.552	0.000	Income (age 23)	-0.961	0.000
Age ('94)	3.297	0.000			
Income ('94)	-0.039	0.000	Income (age 33)	-0.199	0.000
Age ('06)	4.467	0.000			
Income ('06)	0.541	0.000	Income (age 42)	0.349	0.000
Age ('14)	5.347	0.000			
Income ('14)	0.688	0.000	Income (age 50)	0.717	0.000
BMI Level	20.979	1.095	BMI Level	23.645	0.116
BMI Slope	6.103	2.833	BMI Slope	1.929	0.119
BMI Quadratic	-0.822	1.320	BMI Quadratic	-0.186	0.032

LHS = left-hand side; RHS = right-hand side; SED = socioeconomic disadvantage.

For peer review only

Table S2. Coefficient estimates from models of BMI growth, cognitive function, and sociodemographic variables, income excluded.

		United States				United Kingdom			
Outcome	Predictor	Estimate	95%	C.I.	*P*	Estimate	95%	C.I.	*P*
BMI Level	Sex (women)	**-1.572**	**[-1.868,**	**-1.276]**	**< 0.001**	**-0.840**	**[-0.979,**	**-0.701]**	**< 0.001**
	Cognitive function	0.123	[-0.141,	0.387]	0.543	**-0.195**	**[-0.301,**	**-0.089]**	**0.001**
	Sex x Cognitive function	**-0.394**	**[-0.691,**	**-0.097]**	**0.033**	-0.014	[-0.152,	0.123]	0.840
	Youth SED	0.182	[-0.003,	0.367]	0.122	**0.277**	**[0.192,**	**0.363]**	**< 0.001**
	Education	**-0.339**	**[-0.534,**	**-0.143]**	**0.004**	**-0.206**	**[-0.303,**	**-0.110]**	**< 0.001**
BMI Slope	Sex (women)	0.148	[-0.163,	0.459]	0.543	**-0.391**	**[-0.542,**	**-0.239]**	**< 0.001**
	Cognitive function	0.019	[-0.258,	0.296]	0.893	-0.023	[-0.142,	0.096]	0.743
	Sex x Cognitive function	0.257	[-0.049,	0.563]	0.180	-0.118	[-0.270,	0.034]	0.231
	Youth SED	0.209	[0.018,	0.401]	0.096	0.025	[-0.071,	0.122]	0.683
	Education	-0.069	[-0.265,	0.127]	0.627	0.065	[-0.027,	0.157]	0.272
	BMI Level	0.416	[0.012,	0.819]	0.112	0.399	[-0.206,	1.003]	0.294
BMI Quadratic	Sex (women)	0.043	[-0.059,	0.144]	0.567	**0.146**	**[0.098,**	**0.194]**	**< 0.001**
	Cognitive function	0.009	[-0.081,	0.099]	0.893	0.012	[-0.026,	0.050]	0.629
	Sex x Cognitive function	-0.085	[-0.185,	0.016]	0.180	0.020	[-0.030,	0.069]	0.558
	Youth SED	-0.013	[-0.078,	0.052]	0.789	0.027	[-0.004,	0.058]	0.183
	Education	0.014	[-0.059,	0.078]	0.789	-0.025	[-0.053,	0.004]	0.183
	BMI Level	**-0.203**	**[-0.336,**	**-0.071]**	**0.012**	-0.072	[-0.211,	0.067]	0.430
	BMI Slope	**-1.501**	**[-1.724,**	**-1.279]**	**< 0.001**	**-1.041**	**[-1.213,**	**-0.870]**	**< 0.001**
χ2		59.740				254.238			
df		19				6			
CFI		0.996				0.985			
SRMR		0.006				0.015			

Models are latent growth curve models, with latent variables for BMI level, slope, and quadratic slope. The same path diagram was used for both samples, except the US sample was modelled with adjustments for individual ages. All coefficient values are multiple regression coefficients from

For peer review only - http://bmjopen.bmj.com/site/about/guidelines.xhtml

the predictor onto the outcome, except the associations between the three BMI latent variables, which are covariances. P-values are corrected for multiple comparisons using the false discovery rate.

For peer review only

For peer review only - http://bmjopen.bmj.com/site/about/guidelines.xhtml

Table S3. Coefficient estimates from models of BMI growth, cognitive function, and sociodemographic variables, income and education excluded.

		United States				United Kingdom			
Outcome	Predictor	Estimate	95%	C.I.	*P*	Estimate	95%	C.I.	*P*
BMI Level	Sex (women)	**-1.628**	**[-1.922,**	**-1.333]**	**< 0.001**	**-0.926**	**[-1.049,**	**-0.803]**	**< 0.001**
	Cognitive function	-0.060	[-0.306,	0.187]	0.732	**-0.231**	**[-0.321,**	**-0.141]**	**< 0.001**
	Sex x Cognitive function	**-0.400**	**[-0.697,**	**-0.103]**	**0.025**	-0.019	[-0.139,	0.101]	0.810
	Youth SED	**0.274**	**[0.101,**	**0.448]**	**0.010**	**0.343**	**[0.270,**	**0.415]**	**< 0.001**
BMI Slope	Sex (women)	0.146	[-0.163,	0.455]	0.534	**-0.401**	**[-0.546,**	**-0.255]**	**< 0.001**
	Cognitive function	-0.022	[-0.282,	0.238]	0.870	0.026	[-0.085,	0.138]	0.793
	Sex x Cognitive function	0.265	[-0.040,	0.570]	0.151	-0.148	[-0.293,	-0.004]	0.067
	Youth SED	**0.231**	**[0.051,**	**0.412]**	**0.030**	0.018	[-0.068,	0.103]	0.793
	BMI Level	0.386	[-0.017,	0.789]	0.129	**0.330**	**[0.170,**	**0.490]**	**< 0.001**
BMI Quadratic	Sex (women)	0.042	[-0.059,	0.143]	0.565	**0.149**	**[0.102,**	**0.196]**	**< 0.001**
	Cognitive function	0.017	[-0.068,	0.102]	0.749	-0.004	[-0.040,	0.033]	0.849
	Sex x Cognitive function	-0.086	[-0.186,	0.014]	0.151	0.029	[-0.019,	0.077]	0.324
	Youth SED	-0.017	[-0.078,	0.044]	0.732	**0.032**	**[0.004,**	**0.060]**	**0.045**
	BMI Level	**-0.193**	**[-0.326,**	**-0.061]**	**0.016**	-0.058	[-0.114,	-0.001]	0.067
	BMI Slope	**-1.497**	**[-1.718,**	**-1.275]**	**< 0.001**	**-1.076**	**[-1.188,**	**-0.964]**	**< 0.001**
$\chi 2$		58.438				269.375			
df		18				5			
CFI		0.996				0.985			
SRMR		0.006				0.016			

Models are latent growth curve models, with latent variables for BMI level, slope, and quadratic slope. The same path diagram was used for both samples, except the US sample was modeled with adjustments for individual ages. All coefficient values are multiple regression coefficients from the predictor onto the outcome, except the associations between the three BMI latent variables, which are covariances. P-values are corrected for multiple comparisons using the false discovery rate.

For peer review only - http://bmjopen.bmj.com/site/about/guidelines.xhtml

Table S4. Coefficient estimates from models of BMI growth, intelligence, and sociodemographic variables, using participants from all ethnic groups.

		United States				United Kingdom			
Outcome	Predictor	Estimate	95%	C.I.	*P*	Estimate	95%	C.I.	*P*
BMI Level	Sex (women)	**-1.252**	**[-1.554,**	**-0.950]**	**< 0.001**	**-0.823**	**[-0.983,**	**-0.664]**	**< 0.001**
	Cognitive function	0.133	[-0.109,	0.375]	0.387	**-0.177**	**[-0.304,**	**-0.050]**	**0.015**
	Sex x Cognitive function	**-0.719**	**[-0.994,**	**-0.444]**	**< 0.001**	-0.076	[-0.237,	0.084]	0.503
	Youth SED	**0.268**	**[0.111,**	**0.426]**	**0.003**	**0.322**	**[0.226,**	**0.418]**	**< 0.001**
	Education	-0.169	[-0.349,	0.010]	0.129	**-0.169**	**[-0.273,**	**-0.064]**	**0.004**
BMI Slope	Sex (women)	**0.430**	**[0.112,**	**0.748]**	**0.020**	**-0.320**	**[-0.486,**	**-0.154]**	**< 0.001**
	Cognitive function	-0.216	[-0.471,	0.039]	0.179	0.047	[-0.090,	0.184]	0.606
	Sex x Cognitive function	-0.073	[-0.357,	0.210]	0.792	-0.143	[-0.313,	0.026]	0.180
	Youth SED	**0.437**	**[0.276,**	**0.598]**	**0.000**	0.034	[-0.070,	0.138]	0.606
	Education	0.176	[-0.005,	0.358]	0.124	0.016	[-0.090,	0.122]	0.777
	BMI Level	**0.735**	**[0.290,**	**1.180]**	**0.003**	**0.428**	**[0.238,**	**0.618]**	**< 0.001**
BMI Quadratic	Sex (women)	-0.010	[-0.114,	0.095]	0.913	**0.134**	**[0.083,**	**0.185]**	**< 0.001**
	Cognitive function	0.067	[-0.019,	0.154]	0.215	-0.009	[-0.051,	0.033]	0.752
	Sex x Cognitive function	0.014	[-0.080,	0.108]	0.893	0.025	[-0.029,	0.078]	0.503
	Youth SED	**-0.121**	**[-0.175,**	**-0.067]**	**< 0.001**	0.026	[-0.007,	0.058]	0.216
	Education	-0.037	[-0.097,	0.023]	0.333	-0.012	[-0.046,	0.021]	0.598
	BMI Level	**-0.407**	**[-0.555,**	**-0.259]**	**< 0.001**	**-0.084**	**[-0.147,**	**-0.020]**	**0.021**
	BMI Slope	**-1.938**	**[-2.187,**	**-1.689]**	**< 0.001**	**-0.971**	**[-1.100,**	**-0.840]**	**< 0.001**
$\chi 2$		111.284				161.44			
df		31				18			
CFI		0.993				0.990			
SRMR		0.015				0.012			

Models are latent growth curve models, with latent variables for BMI level, slope, and quadratic slope - see the path diagram in Figure 1. The same path diagram was used for both samples, except the US sample was modeled with adjustments for individual ages. All coefficient values are

For peer review only - http://bmjopen.bmj.com/site/about/guidelines.xhtml

multiple regression coefficients from the predictor onto the outcome, except the associations between the three BMI latent variables, which are covariances. P-values are corrected for multiple comparisons using the false discovery rate. SED = socioeconomic disadvantage.

For peer review only

For peer review only - http://bmjopen.bmj.com/site/about/guidelines.xhtml

1. Li W, Kelsey JL, Zhang Z, et al. Small-area estimation and prioritizing communities for obesity control in Massachusetts. *Am J Public Health* 2009;99(3):511-19.
2. Palmer P, Hartke DD, Ree MJ, et al. Armed Services Vocational Aptitude Battery (ASVAB): Alternate Forms Reliability (Forms 8, 9, 10, and 11): AIR FORCE HUMAN RESOURCES LAB BROOKS AFB TX, 1988.
3. Welsh Jr JR, Kucinkas SK, Curran LT. Armed services vocational battery (ASVAB): Integrative review of validity studies: OPERATIONAL TECHNOLOGIES CORP SAN ANTONIO TX, 1990.
4. Der G, Batty GD, Deary IJ. The association between IQ in adolescence and a range of health outcomes at 40 in the 1979 US National Longitudinal Study of Youth. *Intelligence* 2009;37(6):573-80.
5. Wraw C, Deary IJ, Der G, et al. Maternal and offspring intelligence in relation to BMI across childhood and adolescence. *Int J Obesity* 2018 doi: 10.1038/s41366-018-0009-1
6. Herrnstein RJ, Murray C. Bell curve: Intelligence and class structure in American life: Simon and Schuster 2010.
7. Pigeon D. Tests used in the 1954 and 1957 surveys. *The home and the school* 1964:129-32.
8. Douglas JWB. The home and the school: MacGibbon & Kee London 1964.
9. Bridger E, Daly M. Does cognitive ability buffer the link between childhood disadvantage and adult health? *Health Psychol* 2017;36(10):966.
10. Goodman A, Joyce R, Smith JP. The long shadow cast by childhood physical and mental problems on adult life. *Proceedings of the National Academy of Sciences* 2011:201016970.
11. Savalei V. Expected versus observed information in SEM with incomplete normal and nonnormal data. *Psychological methods* 2010;15(4):352.
12. Wraw C, Deary IJ, Gale CR, et al. Intelligence in youth and health at age 50. *Intelligence* 2015;53:23-32.
13. Wraw C, Deary IJ, Der G, et al. Intelligence in youth and mental health at age 50. *Intelligence* 2016;58:69-79.

For peer review only - http://bmjopen.bmj.com/site/about/guidelines.xhtml

Reporting checklist for cohort study.

Based on the STROBE cohort guidelines.

Instructions to authors

Complete this checklist by entering the page numbers from your manuscript where readers will find each of the items listed below.

Your article may not currently address all the items on the checklist. Please modify your text to include the missing information. If you are certain that an item does not apply, please write "n/a" and provide a short explanation.

Upload your completed checklist as an extra file when you submit to a journal.

In your methods section, say that you used the STROBE cohortreporting guidelines, and cite them as:

von Elm E, Altman DG, Egger M, Pocock SJ, Gotzsche PC, Vandenbroucke JP. The Strengthening the Reporting of Observational Studies in Epidemiology (STROBE) Statement: guidelines for reporting observational studies.

		Reporting Item	Page Number
**Title and abstract**			
Title	#1a	Indicate the study's design with a commonly used term in the title or the abstract	1
Abstract	#1b	Provide in the abstract an informative and balanced summary of what was done and what was found	2-3

For peer review only

**Introduction**			
Background / rationale	#2	Explain the scientific background and rationale for the investigation being reported	5-6
Objectives	#3	State specific objectives, including any prespecified hypotheses	6-7
**Methods**			
Study design	#4	Present key elements of study design early in the paper	
Setting	#5	Describe the setting, locations, and relevant dates, including periods of recruitment, exposure, follow-up, and data collection	7-8
Eligibility criteria	#6a	Give the eligibility criteria, and the sources and methods of selection of participants. Describe methods of follow-up.	7-9
Eligibility criteria	#6b	For matched studies, give matching criteria and number of exposed and unexposed	N/A-No matching
Variables	#7	Clearly define all outcomes, exposures, predictors, potential confounders, and effect modifiers. Give diagnostic criteria, if applicable	8
Data sources / measurement	#8	For each variable of interest give sources of data and details of methods of assessment (measurement). Describe comparability of assessment methods if there is more than one group. Give information separately for	8

For peer review only

		for exposed and unexposed groups if applicable.	
Bias	#9	Describe any efforts to address potential sources of bias	8-9
Study size	#10	Explain how the study size was arrived at	7-9
Quantitative variables	#11	Explain how quantitative variables were handled in the analyses. If applicable, describe which groupings were chosen, and why	8-9
Statistical methods	#12a	Describe all statistical methods, including those used to control for confounding	8-9
Statistical methods	#12b	Describe any methods used to examine subgroups and interactions	8-9
Statistical methods	#12c	Explain how missing data were addressed	8-9
Statistical methods	#12d	If applicable, explain how loss to follow-up was addressed	8-9
Statistical methods	#12e	Describe any sensitivity analyses	12-13
**Results**			
Participants	#13a	Report numbers of individuals at each stage of study—eg numbers potentially eligible, examined for eligibility, confirmed eligible, included in the study, completing follow-up, and analysed. Give information separately for for exposed and unexposed groups if applicable.	Table 1

For peer review only

Participants	#13b	Give reasons for non-participation at each stage	N/A-cohort study
Participants	#13c	Consider use of a flow diagram	Cf. Figure 1
Descriptive data	#14a	Give characteristics of study participants (eg demographic, clinical, social) and information on exposures and potential confounders. Give information separately for exposed and unexposed groups if applicable.	Table 1
Descriptive data	#14b	Indicate number of participants with missing data for each variable of interest	Table 1
Descriptive data	#14c	Summarise follow-up time (eg, average and total amount)	9-10, Table 1
Outcome data	#15	Report numbers of outcome events or summary measures over time. Give information separately for exposed and unexposed groups if applicable.	Table 1
Main results	#16a	Give unadjusted estimates and, if applicable, confounder-adjusted estimates and their precision (eg, 95% confidence interval). Make clear which confounders were adjusted for and why they were included	11-12
Main results	#16b	Report category boundaries when continuous variables were categorized	N/A-no variables were made into categories

For peer review only

Main results	#16c	If relevant, consider translating estimates of relative risk into absolute risk for a meaningful time period	N/A-risk not a factor
Other analyses	#17	Report other analyses done—e.g., analyses of subgroups and interactions, and sensitivity analyses	12-13
**Discussion**			
Key results	#18	Summarise key results with reference to study objectives	13-15
Limitations	#19	Discuss limitations of the study, taking into account sources of potential bias or imprecision. Discuss both direction and magnitude of any potential bias.	15-16
Interpretation	#20	Give a cautious overall interpretation considering objectives, limitations, multiplicity of analyses, results from similar studies, and other relevant evidence.	16-17
Generalisability	#21	Discuss the generalisability (external validity) of the study results	15-17
**Other Information**			
Funding	#22	Give the source of funding and the role of the funders for the present study and, if applicable, for the original study on which the present article is based	18

None The STROBE checklist is distributed under the terms of the Creative Commons Attribution License CC-BY. This checklist can be completed online using https://www.goodreports.org/, a tool made by the EQUATOR Network in collaboration with Penelope.ai

For peer review only
